# Distinct neural signatures in a sensorimotor synchronization-continuation task

**DOI:** 10.1162/IMAG.a.1100

**Published:** 2026-01-14

**Authors:** Dae-Jin Kim, Amanda R. Bolbecker, Alexandra B. Moussa-Tooks, Krista M. Wisner, Brian F. O’Donnell, Emily L. Gildea, William P. Hetrick

**Affiliations:** Department of Psychological & Brain Sciences, Indiana University, Bloomington, IN, United States; Program of Neuroscience, Indiana University, Bloomington, IN, United States; Department of Psychiatry, Indiana University School of Medicine, Indianapolis, IN, United States

**Keywords:** internal model, sensorimotor synchronization, finger tapping, synchronization, continuation

## Abstract

Optimal sensorimotor timing hinges on the generation, refinement, and employment of internal models to meet task demands. In finger tapping sensorimotor synchronization tasks, this occurs across and within tapping conditions that prompt externally-cued synchronization, followed by un-cued continuation. Theory suggests within each condition, initial behavioral performance is adjusted by internal models. However, whether distinct within- and between condition subprocesses are associated with activation of unique neural networks remains unknown. During fMRI, 100 neurotypical adults completed a finger tapping task with synchronization and continuation conditions. Rapid improvement in tapping accuracy occurred during the initial seconds of both synchronization and continuation conditions. Tapping performance in the first few seconds of each condition was marked by heightened functional activity across sensorimotor, prefrontal-parietal-temporal, and salience network regions compared to subsequent within-condition performance, suggesting rapid refinement of an internal model. Intensity of functional activity within the same regions correlated with task performance. Findings highlight dynamic processes supporting development and refinement of internal models for sensorimotor timing.

## 1 Introduction

Many cognitive and behavioral processes rely on our ability to engage effectively with the environment, with optimal timing playing a central role. This involves developing accurate internal models to guide actions. Such timing is crucial not only for sensorimotor tasks but also for social functioning, motor skills, perception, and cognitive processes like word generation, attention, and working memory. A putative internal model is crucial for performance in sensorimotor timing tasks, such as sensorimotor synchronization (Wolpert et al., 1995). These tasks involve *synchronization conditions*, where rhythmic sensory stimuli (e.g., auditory or visual) are coordinated with motor actions like finger tapping (i.e., tone-paced tapping; Pressing, 1999; Repp, 2005; Repp & Su, 2013), and *continuation conditions*, where rhythmic motor actions continue without external cues (i.e., self-paced tapping condition). During synchronization, the brain relies on an internal model to time motor responses using sensory input. This model enables anticipatory motor responses, as evidenced by taps preceding tones by a few milliseconds (Repp, 2005). Once established, the internal temporal model is used during continuation conditions to maintain timing in the absence of external stimuli. Generation and optimization of the internal model of movement is a dynamic process (Shadmehr & Mussa-Ivaldi, 1994). The dynamic process involves the memory of the stimulus period (i.e., temporal rate of the tone), which interacts with sensorimotor synchronization mechanisms. During synchronization, corrective mechanisms adjust tapping timing to align with external cues while establishing an “internal timekeeper” based on the interstimulus intervals (Repp & Su, 2013). In continuation, this internal model is executed and adaptively corrected to maintain accuracy, relying on memory of the period to sustain synchronization in the absence of external cues.

Synchronization is a highly optimized process, achieving a stable phase trajectory after only a few external cues (Fraisse, 1966). Evidence suggests that updating the internal model involves distinct behavioral and neural mechanisms (Flach, 2005; Kroger et al., 2021; Merchant et al., 2011; Witt et al., 2008). While motor actions during synchronization rely on both external cues and the internal model, the degree of reliance on external cues, and the functional involvement across neural regions, may be shifted—for example, from the synchronization to continuation condition (Lewis et al., 2004). Cortical areas (e.g., temporal and pre/post-central gyri, lateral prefrontal cortex) and subcortical regions (cerebellum, basal ganglia) support functions like timing detection, motor coordination, attention, and error correction (Gamez et al., 2019; Harry et al., 2023; Jantzen et al., 2007; Merchant et al., 2011; Penhune & Zatorre, 2019). Despite extensive research, how sensorimotor timing varies within early and late phases of synchronization and continuation conditions, considering dynamic error correction for internal models, remains unclear. Additionally, the neural networks underlying distinct timing processes within and between conditions are not well understood. In this study, participants performed a sensorimotor synchronization-continuation finger-tapping task. Our goals were to test whether sensorimotor timing varies between the initial and late phases of each condition and to identify neural substrates associated with internal modeling and updating processes (Kim et al., 2015; Makino et al., 2016; Wolpert & Flanagan, 2016). Given the task’s simplicity, we examined dynamic tapping behaviors every second to capture time-varying characteristics.

Motor learning in repetitive synchronization tasks has been conceptualized using several frameworks, including the two-stage model with fixation and diversification stages (Gentile, 1972) and the three-stage models – cognitive, associative, and autonomous stages (Fitts & Posner, 1967) and the freezing, releasing, and exploiting stages (Bernstein, 1996). While the three-stage models emphasize cognitive automatization, Gentile’s model focuses on task–environment adaptation, which is particularly relevant to sensorimotor synchronization under constant rhythmic conditions, as examined in the present study. Therefore, we first hypothesized that in the early seconds of synchronization—characterized by greater tapping errors during the initial timing process—there would be a higher functional demand to construct and refine an internal model capable of accurately encoding predictions for the desired motor output. Sensorimotor synchronization is known to engage multiple brain regions and involves both predictive and reactive control mechanisms, with the early phases (e.g., the initial few seconds within a tapping condition) requiring more active engagement of specific neural circuits (Repp & Su, 2013). We anticipated greater functional brain activation in these early phases of tapping conditions, particularly in the sensorimotor cortex, basal ganglia, and anterior cerebellar regions (Witt et al., 2008). As internal model formation follows a first-order exponential convergence toward a state determined by initial conditions and target sequences (Pierella et al., 2019), and as timing mechanisms become increasingly efficient as the internal model is refined (Paton & Buonomano, 2018), we expected functional involvement to diminish once the internal model (timekeeper) is established and errors minimized. During this later stage of synchronization, we anticipated that less effortful and largely unconscious adjustments of motor execution would predominate, ensuring that tapping remains aligned with the internal model and external cues. Second, we hypothesized that continuation tapping – maintaining tapping once external cues have ceased – would result in immediate fluctuations in tapping accuracy due to the abrupt cessation of tone-driven tempo and the subsequent need to adjust the established internal model. This hypothesis is supported by evidence that continuation tapping relies on distinct internal timekeeping mechanisms and self-monitoring processes compared to synchronization with external stimuli (Repp & Su, 2013). Additionally, the sudden loss of external cues necessitates reactivation and adjustment of internal models to sustain performance (Kawato, 1999). In the absence of external cues, the brain relies more heavily on internal timing mechanisms and engages in active error correction to maintain motor performance (Leow & Grahn, 2014). This supports the expectation of heightened functional involvement during the early phase of the continuation condition, as the system adjusts to the lack of external pacing. Thus, compared to synchronization, we anticipated greater overall functional activity during continuation due to the increased demands of self-monitoring and the reapplication and adjustment of the internal model to maintain accurate tapping. As in the synchronization condition, we anticipated that functional involvement would evolve throughout the continuation condition, with greater correction of motor timing occurring in the early seconds and diminishing as errors are minimized through iterative refinement. Our final hypothesis posits that higher brain activity in specific regions would correlate with improved tapping accuracy and reduced errors during sensorimotor synchronization tasks. Prior research suggests that increased neural activity in certain brain regions (e.g., primary/supplementary motor regions including premotor cortex, supplementary motor area, and dorsolateral prefrontal cortex) is associated with enhanced motor accuracy and complexity (Anwar et al., 2016; Lewis et al., 2004; Luft et al., 2020). Based on this evidence, we tested whether decomposition of the time-course of brain activity could identify key regions linked to behavioral metrics, including tapping accuracy, across synchronization and continuation conditions. We anticipated that higher brain activity would be associated with better tapping accuracy and less tapping error, reflecting increased functional involvement in task performance.

## 2 Materials and Methods

### 2.1 Participants inclusion/exclusion criteria

The study protocol was approved by Indiana University’s Institutional Review Board (Protocol numbers: 1812741373 and 1902526170), and all participants provided written informed consent. Participants, aged 18–39, were recruited from Bloomington, Indiana, meeting criteria for normal hearing and vision, limited alcohol consumption, no illicit substance use, and no psychiatric history. All participants were non-musicians with no formal musical training. No one experienced any exclusion criteria, which included contraindications for MRI, IQ < 80 (WASI: Wechsler Abbreviated Scale of Intelligence), psychiatric or neurological conditions (e.g., epilepsy, bipolar disorder), pregnancy, or implanted devices. Twenty-six participants were additionally excluded due to the excessive movement during fMRI (N = 13, Supplementary Fig. S1), unusable tapping data (N = 11, Supplementary Fig. S2), and a mechanical fault during data transfer (N = 2), respectively. The final dataset comprised 100 neurotypical adults. Demographics are detailed in Table 1.

**Table 1.** Demographic characteristics of the participants.

Number of participants		100
Male/female		47/53
Age (years)		22.86 ± 4.29
		18–39
Handedness (right/left/ambidextrous/unknown)		90/8/1/1
Intelligence quotient (IQ)	Full scale IQ-2 (FSIQ-2)	113.76 ± 9.58
		92–137
	Vocabulary	58.67 ± 7.56
		35–80
	Matrix reasoning	56.96 ± 7.96
		34–75
Digit symbol substitution test		12.37 ± 2.58
		7–19
Letter-number sequencing		21.20 ± 3.63
		11–29

The table presents the group means (±standard deviations) and ranges (minimum-maximum). IQ was measured by WASI-2 (Wechsler Abbreviated Scale of Intelligence | Second Edition). Handedness was measured by the modified version of Edinburgh Handedness Inventory (Oldfield, 1971).

### 2.2 Experimental paradigm

**Fig. 1.**
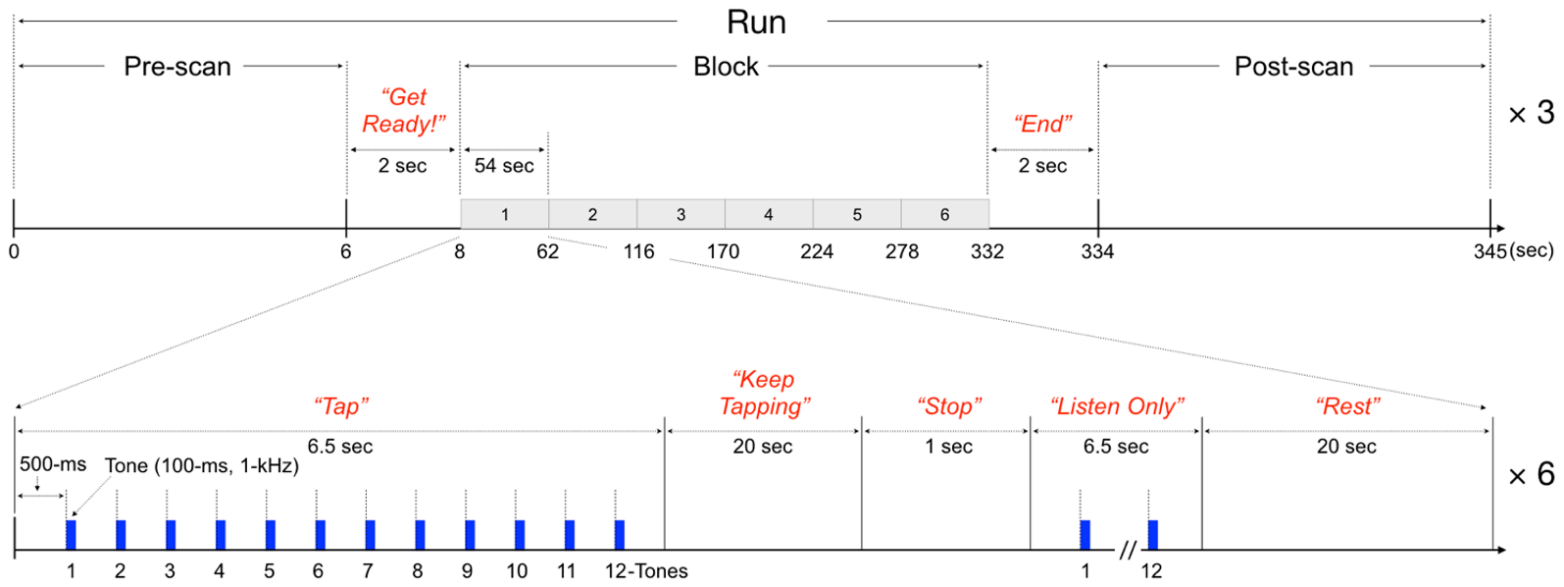
Finger-tapping synchronization-continuation paradigm for fMRI. The finger-tapping task consisted of three successive runs, each divided into the following phases: pre-scan (6 sec; for MR stabilization), task preparation (2 sec), the main task block (324 sec), task ending (2 sec), and post-scan acquisitions (11 sec; technical buffer for imaging completeness). Each main task block comprised six trials, with each trial following this sequence: synchronization (tone-paced tapping, where participants tapped in sync with a tone for 6.5 sec), continuation (self-paced tapping without the tone for 20 sec), stop instruction (1 sec), listening (passive tone listening without tapping for 6.5 sec), and resting (20 sec). During the synchronization and listening phases, 12 tones were presented, each lasting 100 ms with a frequency of 1 kHz and an inter-onset interval (IOI) of 500 ms.

The sensorimotor synchronization-continuation task used in this study was adapted from our previous fMRI study (Moussa-Tooks et al., 2019). Participants performed a sensorimotor synchronization finger-tapping task during fMRI scanning, consisting of three runs (Fig. 1). Each run included six trials of synchronization (tone-paced tapping, 6.5 sec), continuation (self-paced tapping, 20 sec), stop instruction (1 sec), listening (6.5 sec), and resting (20 sec). Participants tapped on an MR-compatible pad measuring tapping pressure. Synchronization involved 12 tones (100 ms, 1 kHz) with a 500 ms inter-onset interval, prompting tone-paced tapping. During continuation, participants tapped at the same rate without tones.

### 2.3 MRI acquisition

MRI data were acquired using a Siemens 3T MAGNETOM Trio-Tim scanner with a 64-channel head coil. T1-weighted (T1w) and T2-weighted (T2w) scans served as anatomical references, while T2*-weighted functional scans were collected using an echo-planar imaging (EPI) sequence. The high-resolution T1w scan used an inversion recovery spoiled gradient recalled acquisition (IR-SPGR) sequence (TR = 2.4 sec, TE = 2.68 ms, voxel size = 0.8 × 0.8 × 0.8 mm³), and the T2w scan used a turbo spin echo (TSE) sequence (TR = 3.2 sec, TE = 564 ms, voxel size = 0.8 × 0.8 × 0.8 mm³). Functional scans were acquired with EPI (TR = 1.0 sec, TE = 30 ms, voxel size = 2 × 2 × 2 mm³, multi-band factor = 6, dimension = 108 × 108 × 72) and included 345 volumes per run (~6 min). With these multiband EPI parameters, slice acquisition occupied nearly the full 1.0-sec TR, and the scanner acoustic noise did not contain a salient silent gap at the end of each volume that could act as an additional rhythmic auditory cue. All anatomical and functional imaging volumes were positioned to ensure full brain coverage, including the cerebellum and the superior cortical regions. Participants viewed task instructions via a mirror and heard auditory tones through headphones, with instructions presented using E-Prime software (https://pstnet.com/products/e-prime, v2.0.10.242).

### 2.4 Computation of inter-tap interval (ITI) and tapping force

**Fig. 2.**
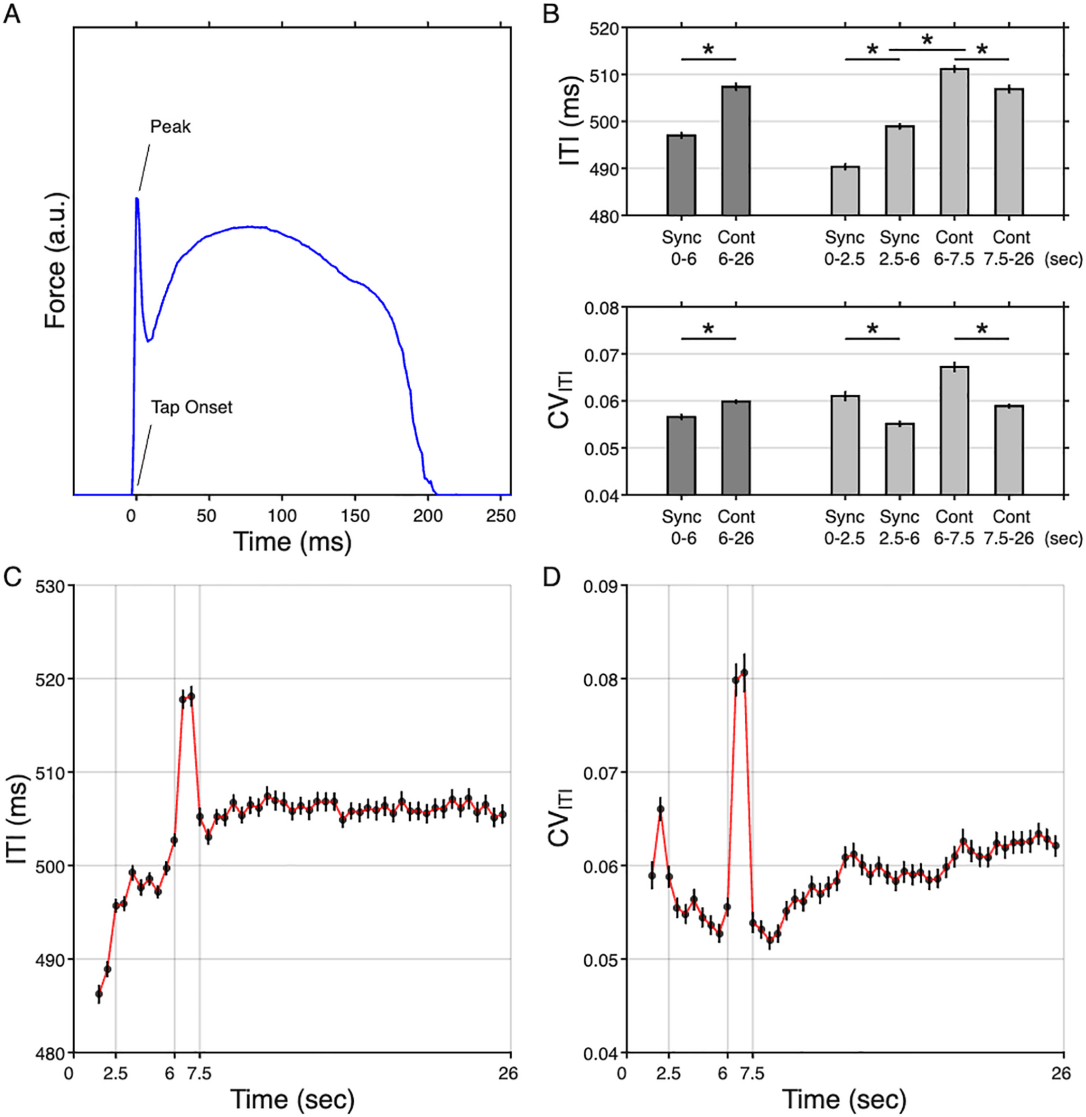
Analysis of tapping behavior. (A) An example of acquired finger tapping signal. (B) Descriptive statistics of inter-tap interval (ITI) and its coefficient of variation (CV_ITI_) averaged for each condition (dark gray) and phase (light gray). Statistical significance (*) represents *p* < 0.05 from repeated-measures ANOVA tests. (C-D) Temporal dynamics of ITI and CV_ITI_. The red and blue lines represent curves derived from the sliding-window moving averaging (SMA) and single tap-based averaging methods, respectively.

The tapping signal was measured using a flat force sensor (FSR03; https://www.ohmite.com) detecting the pressure of a right index finger applied to a force membrane sensor, which converts the tapping force to a relative voltage output to the baseline (Fig. 2A). The peak and its onset time of each tapping were defined at the point where tapping pressure reached at the maximum force, considering the negligible duration between the rising instant from the baseline and the actual peak onset (<5 ms). Note that there are two peaks—the first, sharp peak is due to impact while the second, broader peak (~200 ms) is related to finger inertia and viscoelastic properties of the pulp (Billon & Semjen, 1995; Jindrich et al., 2003; Rempel et al., 1994). The first tap was excluded as it reflects reaction rather than synchronization. Upon carefully inspecting the tapping behavioral patterns, we identified that 4 participants each had at least one unusable trial out of the total 18 trials (3 runs × 6 trials per run), resulting in a total of 24 excluded trials. Inter-tap interval (ITI) and tapping force during synchronization and continuation were measured to characterize finger-tapping behavior (Bolbecker et al., 2011; Carroll et al., 2009; Moussa-Tooks et al., 2019). The rationale for including force was to capture aspects of motor execution beyond timing accuracy. While ITI quantifies temporal synchronization, tapping force reflects the intensity and stability of motor output, which are also critical components of sensorimotor control. Also, it serves as an additional quality-control indicator, as excessively weak or highly inconsistent force may suggest insufficient task engagement or motor difficulty. Coefficient of variation (CV) was calculated for ITI and force to assess variability. Peak tapping force was defined as the maximum pressure within 125 ms of tapping onset. Force values below 500 a.u. were excluded as atypical, and high-pass filtering (>10 Hz) minimized redundant fluctuations. ITI was calculated as the interval between peaks of successive taps, excluding values >1000 ms (missed taps) or <250 ms (fast taps) (Helmuth & Ivry, 1996). Refer to Supplementary Figure S2 for unusable tap exclusions.

### 2.5 Time-varying characteristics of tapping measures

To capture dynamic sensorimotor synchronization over short timescales, tapping measures were analyzed using a sliding-window moving average (SMA) method. This approach computed averages within a 2-sec window (4–5 taps), incremented every 500 ms across the 26-sec synchronization and continuation period. Measurements were divided into equal-sized segments, and SMA values were calculated as the mean within each window. Specifically, let *x* = {*x_i_* | 0 < *x_i_* ≤ *T*, *i* = 1, 2, ⋯, *N_x_*} represent a tapping measure (e.g., ITI or tapping force), where *T* was set to 26 sec of the synchronization and continuation period, and *N_x_* was the number of measurements. With SMA method, we divided the measurements, *x*, into several consecutive equal-sized segments (*w*) defined by *w_t(n)_* = {*x_k_* | *t(n)*-*W* ≤ *x_k_* ≤ *t(n)*+*W*, *k* = 1, 2, ⋯, *N_t(n)_*}, where *t*(*n*), *W*, and *N_t(n)_* represent the *n*-th offset time for shifting the window – *t*(*n*) = {1, 2, ⋯, 26}), the window size in seconds, and the number of measurements within the window at *t*(*n*), respectively. The moving averaged measurement within the window at *t*(*n*) was computed as *x_SMA,t_*_(_*_n_*_)_ = Σ*w_t_*_(_*_n_*_)_/*N_t_*_(_*_n_*_)_. Additional results for varying window sizes are shown in Supplementary Figure S3. As a methodological reference, we additionally compared the SMA method to a more conventional event-based averaging approach, in which ITI and CV were calculated at each tap based on the tapping order and assigned to the corresponding tone onset—that is, the participant’s n-th tap was aligned with the n × 500 ms time point.

### 2.6 Statistical analysis of tapping behaviors

To examine differences in tapping behaviors between synchronization and continuation, we first regressed out the effects of age and sex across all participants, then a repeated-measures ANOVA was conducted with three within-subjects factors: condition (synchronization and continuation), run (1, 2, and 3), and trial (1, 2, 3, 4, 5, and 6). If significant differences between the early and late phases of each measure existed, a subsequent repeated-measures ANOVA with four factors—condition, phase (early and late), run, and trial—was performed to further analyze these effects. Statistical significance was assessed using the Tukey-Kramer method for multiple comparison correction at *p* < 0.05.

### 2.7 MRI preprocessing

Raw MRI data were converted to Brain Imaging Data Structure (BIDS) format using HeuDiConv (https://heudiconv.readthedocs.io, v0.11.6). The initial assessment of data quality was performed using MRIQC (https://mriqc.readthedocs.io, v0.16.1; with despike, slice-timing correction, ica options) and two unusable participant datasets due to data loss were found and excluded at this step. Subsequent preprocessing of the functional and anatomical MRIs was carried out using fMRIPrep (https://fmriprep.org, v20.2.5), which included estimation of motion parameters, slice-timing correction, motion correction, coregistration, spatial normalization to MNI152NLin2009cAsym template, removal of non-steady state volumes, spatial smoothing (isotropic Gaussian kernel of 6 mm FWHM), and removal of ICA-identified motion artifacts (ICA-AROMA). The fMRIPrep boilerplate is provided in the Supplementary Material.

### 2.8 Statistical analysis of fMRI

FEAT (FMRI Expert Analysis Tool, v6.00) of FSL (FMRIB Software Library, https://fsl.fmrib.ox.ac.uk) was employed for all fMRI statistical analyses. Initially, a subject-level analysis with a General Linear Model (GLM) was conducted for each participant, encompassing brain extraction, temporal high-pass filtering (>90 sec), FILM (FMRIB’s Improved Linear Model) prewhitening, double-gamma hemodynamic response function (HRF), inclusion of the temporal derivatives of HRF-convolved regressors, and temporal filtering to the model. Additionally, age, sex, and framewise displacement (FD) of each participant were included as confounding variables. Six task-related regressors were empirically derived based on the temporal dynamics of the group-averaged ITI and coefficient of variation of ITI (CV_ITI_): early synchronization (Sync1: 0–2.5 sec), late synchronization (Sync2: 2.5–6 sec), early continuation (Cont1: 6–7.5 sec), late continuation (Cont2: 7.5–26.5 sec), stop and listen (26.5–34 sec), and rest (34–54 sec) (see Section 3.2 and Fig. 2C-D). These regressors were fitted to the main task blocks at the first level (324 volumes). Subsequently, the following contrasts were entered: Synchronization (=Sync1-Rest, Sync2-Rest, and Sync1-Sync2), Continuation (=Cont1-Rest, Cont2-Rest, and Cont1-Cont2), and Synchronization *vs*. Continuation (=Sync1-Cont1 and Sync2-Cont2). Since no significant activation was observed outside the temporal cortex during listen in our previous study (Moussa-Tooks et al., 2019), the regressor was excluded from the contrast of interest. At the second level, contrast images were averaged across three runs for each participant using a fixed-effect analysis. For the group-level analysis, the repeated-measures analysis with FLAME 1 (FMRIB’s Local Analysis of Mixed Effects) was employed across all participants to obtain accurate parameter estimates by incorporating information from both within- and between-subject variability. All statistical results were thresholded at *Z* > 3.1, equivalent to *p* < 0.05 at cluster level, and whole-brain clusters were corrected for multiple comparisons at *p* < 0.05.

### 2.9 Brain-behavior correlation

We subsequently computed single-subject level correlations between the time-resolved fMRI parameter estimates and tapping behaviors (ITI, force, and their CVs). To examine the temporal fluctuation of functional activations on BOLD activity, we utilized a finite impulse response (FIR) model using FEAT’s FIR basis convolution. Four task conditions were modeled as separate regressors: synchronization (tone-paced), continuation (self-paced), listen, and rest. Synchronization and continuation were modeled using an FIR basis set with a 26-sec window (6.5-sec synchronization followed by 20-sec continuation) time-locked to the onset of the synchronization block, with 26 1-sec bins (regressors). The listen and rest periods were additionally modeled as boxcar regressors of 7.5 sec and 20 sec duration, respectively, and were treated as regressors of no interest to account for variance during non-tapping periods. This allowed the estimation of the hemodynamic response over time for tone- and self-paced tapping tasks while capturing possible temporal variations in peak latency or amplitude across brain regions duration after the onset of stimulus. High-pass temporal filtering (90 sec cutoff) and prewhitening were applied as implemented in FSL. All regressors were orthogonalized within conditions and convolved with delta functions corresponding to the FIR bins. Parameter estimates (β-values) for each 1-sec time bin were obtained for every voxel, providing time-resolved BOLD responses for each condition. These estimates for each run were then used for subsequent brain–behavior correlation analyses with tapping performance metrics (ITI and CV). The fMRI parameter estimates were upsampled to a 0.5 sec sampling rate using Matlab’s *interp1* function for linear interpolation (MathWorks, R2023b) and correlated with the temporal fluctuation of the tapping measurements extracted from the SMA method at each voxel, considering the delay of hemodynamic response function (HRF)—from 0 to 6 sec. In our analyses, ITI was converted to the absolute value of the deviation from the 0.5 sec of inter-tone interval, and we used absolute value of the *z*-scored force, respectively. Higher values were interpreted as worse tapping behaviors, indicating more deviation in tapping interval, force, and their consistency. The correlations were computed for ITI, force, and CVs of ITI and force, respectively. We used *Randomise*, an FSL tool that extends the permutation testing framework to allow user-defined statistical designs (Winkler et al., 2014), which included covariates for age, sex, and head motion. Voxel-wise statistics were computed on correlation maps averaged across runs for each participant, using 10,000 permutations within a general linear model to assess significant brain–behavior associations across HRF delays. Multiple comparisons were corrected using threshold-free cluster enhancement (TFCE), and results surviving family-wise error (FWE) correction at *p* < 0.05 were visualized on surface maps in standard space.

## 3 Results

### 3.1 Descriptive analysis of tapping behavior

Inter-tap interval (ITI), tapping force, and their coefficients of variation (CV) were analyzed to assess tapping behavior. ITI (interval between peaks of successive taps) and force (tapping pressure) consistency were measured using CV (standard deviation/mean). A repeated-measures ANOVA with 3 within-subjects factors (condition, run, and trials) revealed a significant main effect of condition on ITI—*F*(1, 3566)=76.67, *p* < 0.001—with synchronization (496.95 ± 32.01 ms) demonstrating faster response times compared to continuation (507.36 ± 38.52 ms) in Figure 2B. The main effect of condition was also found on CV_ITI_—*F*(1, 3566)=14.16, *p* = 0.002—with less tapping variance in synchronization (0.057 ± 0.029) compared to continuation (0.060 ± 0.022). The main effect of condition was not significant for the force measures (all *ps* > 0.05). A significant main effect of trial was observed on ITI, *F*(5, 3566)=3.64, *p* < 0.003, such that the fourth trial showed a longer ITI (507.67 ± 86.69 ms) compared with the other trials (approximately 500–502 ms on average). Although this effect reached significance, there was no interaction between condition and trial (*p* = 0.70), indicating that it did not influence the primary experimental comparison. No significant main effects of run or trial were found for CV_ITI_ or for the force measures (Force and CV_Force_).

### 3.2 Distinct temporal dynamics of tapping behavior

Time-varying behaviors were calculated with a sliding-window moving average (SMA; ±1 sec, every 500 ms) and shown in Figure 2C-D and Supplementary Figure S4. ITI increased during the first 2.5 sec of synchronization as CV decreased, stabilizing thereafter, highlighting behaviorally distinct early (Sync1; 0–2.5 sec) and late (Sync2; 2.5–6 sec) phases. After tones stopped at 6 sec, ITI and CV_ITI_ fluctuated (Cont1; 6–7.5 sec) and stabilized (Cont2: 7.5–26 sec), suggesting a reconstructed tapping strategy, while tapping force showed no distinct patterns (Supplementary Fig. S4C-D). A repeated-measures ANOVA with 4 factors (condition, phase, run, and trials) revealed a significant main effect of phase on ITI—*F*(1, 7126)=6.37, *p* = 0.012—with the early phase (500.76 ± 37.09 ms) demonstrating faster response times compared to the late phase (502.90 ± 35.82 ms). The interaction effect between condition and phase was significant—*F*(1, 7101)=58.61, *p* < 0.001—indicating that the effect of condition on ITI varied across phases. Specifically, ITI during early synchronization was significantly shorter than during late synchronization (Sync1: Sync2 = 490.34 ± 34.44: 498.94 ± 31.26, *p* < 0.001). Conversely, ITI during early continuation was significantly longer than during late continuation (Cont1: Cont2 = 511.16 ± 36.72: 506.87 ± 39.46, *p* = 0.001; Fig. 2B). The main effect of phase was also found on CV_ITI_—*F*(1, 7126)=59.34, *p* < 0.001—with higher tapping variance in the early phase (0.064 ± 0.049) compared to the late phase (0.057 ± 0.027). The main effect of phase was not significant for the force measures (all *ps* > 0.05). Since the force measured did not reveal distinct changes over conditions and phases (Supplementary Fig. S4), subsequent analyses were reported only for ITI and CV_ITI_ measures.

### 3.3 Functional activation of early and late synchronization

**Fig. 3.**
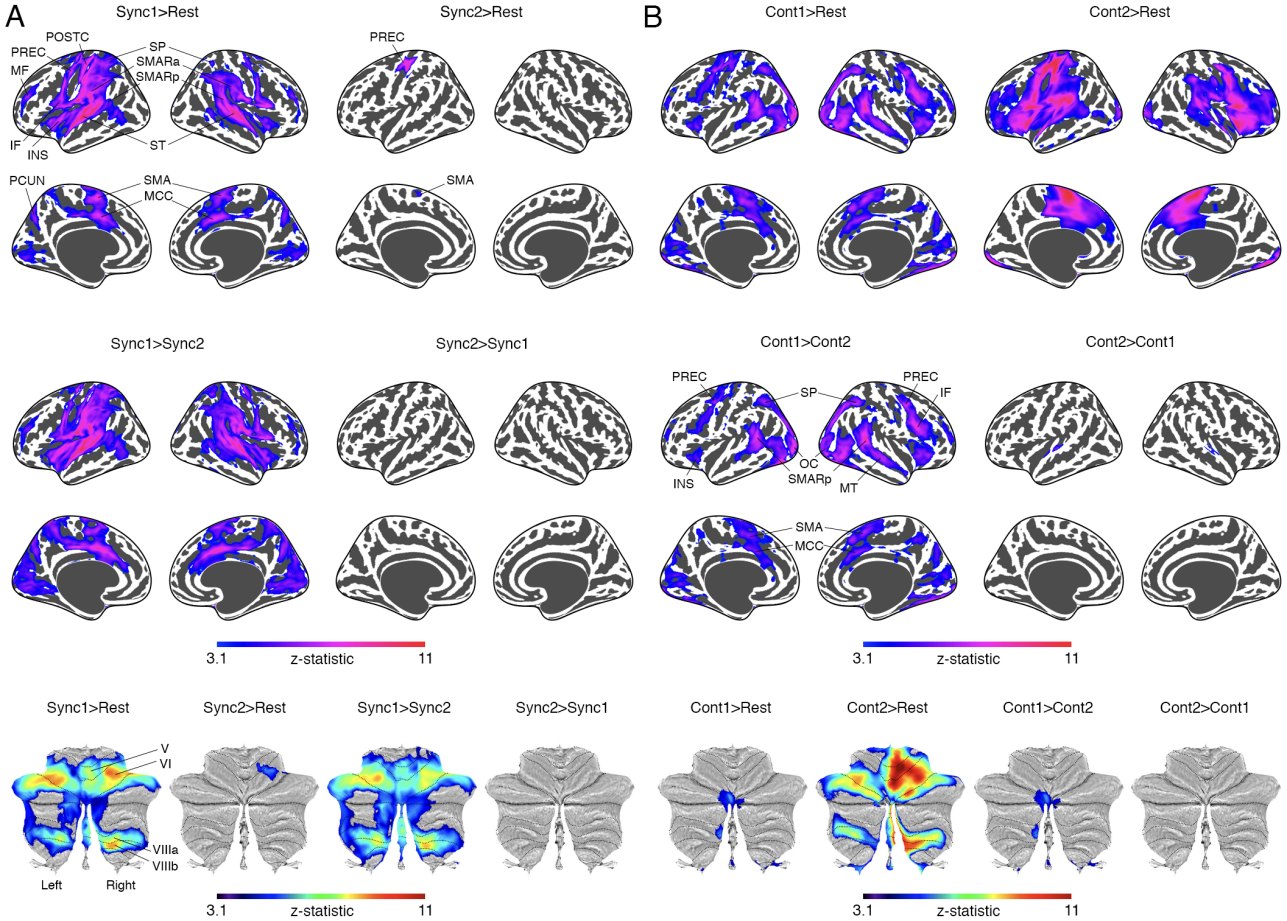
Functional activation of synchronization and continuation. (A) Functional activation of early (Sync1) and late (Sync2) synchronization. (B) Functional activation of early (Cont1) and late (Cont2) continuation. Early and late phases of synchronization and continuation were defined based on behavioral analysis (Fig. 2). Significant results were set at *p* < 0.001 (voxel level) and *p* < 0.05 (cluster level). See the peak locations in Supplementary Tables S1-S2. *Abbreviation*. IF, inferior frontal; INS, insula; MCC, middle cingulate; MF, middle frontal; MT, middle temporal; OC, occipital cortex; PCUN, precuneus; POSTC, postcentral; PREC, precentral; SMA, supplementary motor area; SMARa/p, anterior/posterior supramarginal; SP, superior parietal; ST, superior temporal.

Using tapping behaviors (Sync1-2 and Cont1-2), we examined neural correlates of sensorimotor timing with a GLM approach in fMRI data from 100 participants. In Figure 3A and Supplementary Table S1, the early tone-paced phase (Sync1 > Rest) showed widespread activation in the sensorimotor network (e.g., primary and supplementary motor areas, somatosensory cortex), prefrontal-parietal-temporal network (e.g., middle/inferior frontal, superior/inferior parietal, and auditory cortex), salience network (insula, anterior-posterior cingulate cortices), occipital cortex, subcortical structures (putamen, amygdala, thalamus), and cerebellum (lobules V, VI, VIIIa/b). In contrast, late tone-paced tapping (Sync2 > Rest) had limited activation in the left precentral/postcentral regions, SMA, and right cerebellar lobule VI. Sync1 showed greater activation than Sync2 (Sync1 > Sync2), but no significant activation was observed for Sync2 > Sync1.

### 3.4 Functional activation of early and late continuation

The early and late phases of continuation showed distinct functional activation patterns during self-paced tapping without auditory tones (Fig. 3B, Supplementary Table S2). The early phase (Cont1 > Rest) exhibited activation in the primary/supplementary motor regions, middle temporal, inferior frontal (right hemisphere), insula, middle cingulate (Palomero-Gallagher et al., 2009), superior parietal, and occipital cortices, with additional subcortical (putamen, thalamus) and cerebellar (Vermis VI, VIIIa/b, X) activations. The late phase (Cont2 > Rest) showed activation in similar regions, including the superior temporal cortex and cerebellum (lobules V, VI, VIIIa/b). Early-phase activation (Cont1 > Cont2) was higher in motor, sensory, temporal, and parietal regions, as well as the thalamus and cerebellum. Late-phase activation (Cont2 > Cont1) was greater only in the superior temporal cortex.

### 3.5 Comparison of each timing phase

**Fig. 4.**
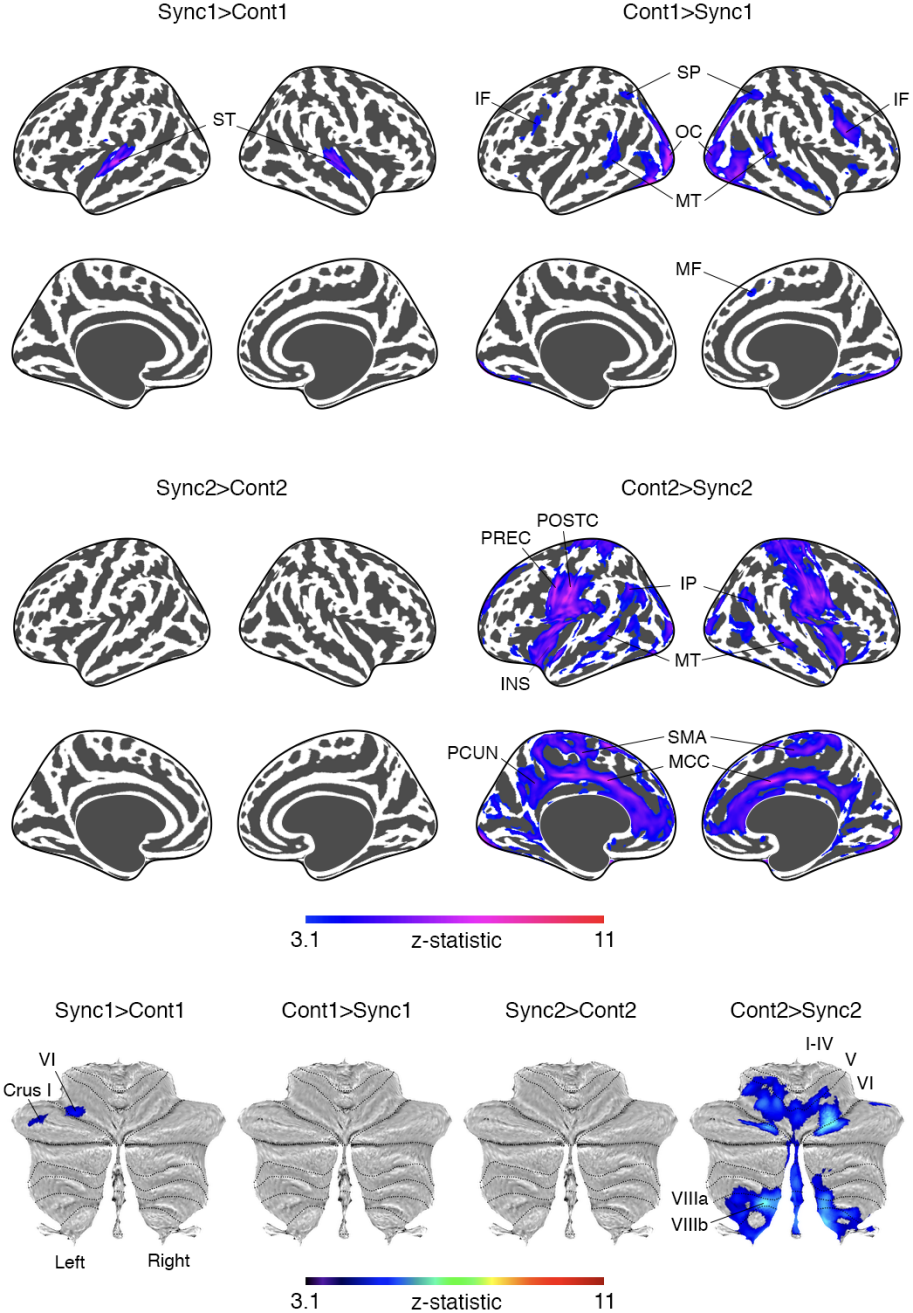
Comparison of each phase for synchronization and continuation. Results were significant at *p* < 0.001 (voxel level) and *p* < 0.05 (cluster level) (See Supplementary Table S3). *Abbreviation*. IF, inferior frontal; INS, insula; IP, inferior parietal; MCC, Middle cingulate; MF, medial frontal; MT, middle temporal; OC, occipital cortex; PCUN, precuneus; POSTC, postcentral; PREC, precentral; SMA, supplementary motor area; ST, superior temporal.

We compared each phase (early and late) of synchronization and continuation to further demonstrate the distinct patterns of functional activation with and without auditory tones (Fig. 4, Supplementary Table S3). The early phase of tone-paced tapping showed higher activation than that of self-paced tapping (Sync1 > Cont1) only in the bilateral superior temporal cortex and in cerebellar lobules VI and Crus I. On the other hand, early phase of self-paced tapping exhibited higher activation compared to the tone-paced one (Cont1 > Sync1) in the prefrontal-parietal-temporal network, including the inferior/medial frontal, superior parietal, middle temporal, and occipital cortices, plus a portion of the right anterior insula and rostral cingulate cortices (Jocham et al., 2009). For the late phase, activation for Cont2 > Sync2 was found in the sensorimotor network (primary/supplementary motor), salience network (insular and cingulate cortices), middle temporal, inferior parietal cortices, and cerebellar activations in the lobules VI and VIIIa/b. No activated region was found for the contrast Sync2 > Cont2.

### 3.6 Brain-behavior correlation

**Fig. 5.**
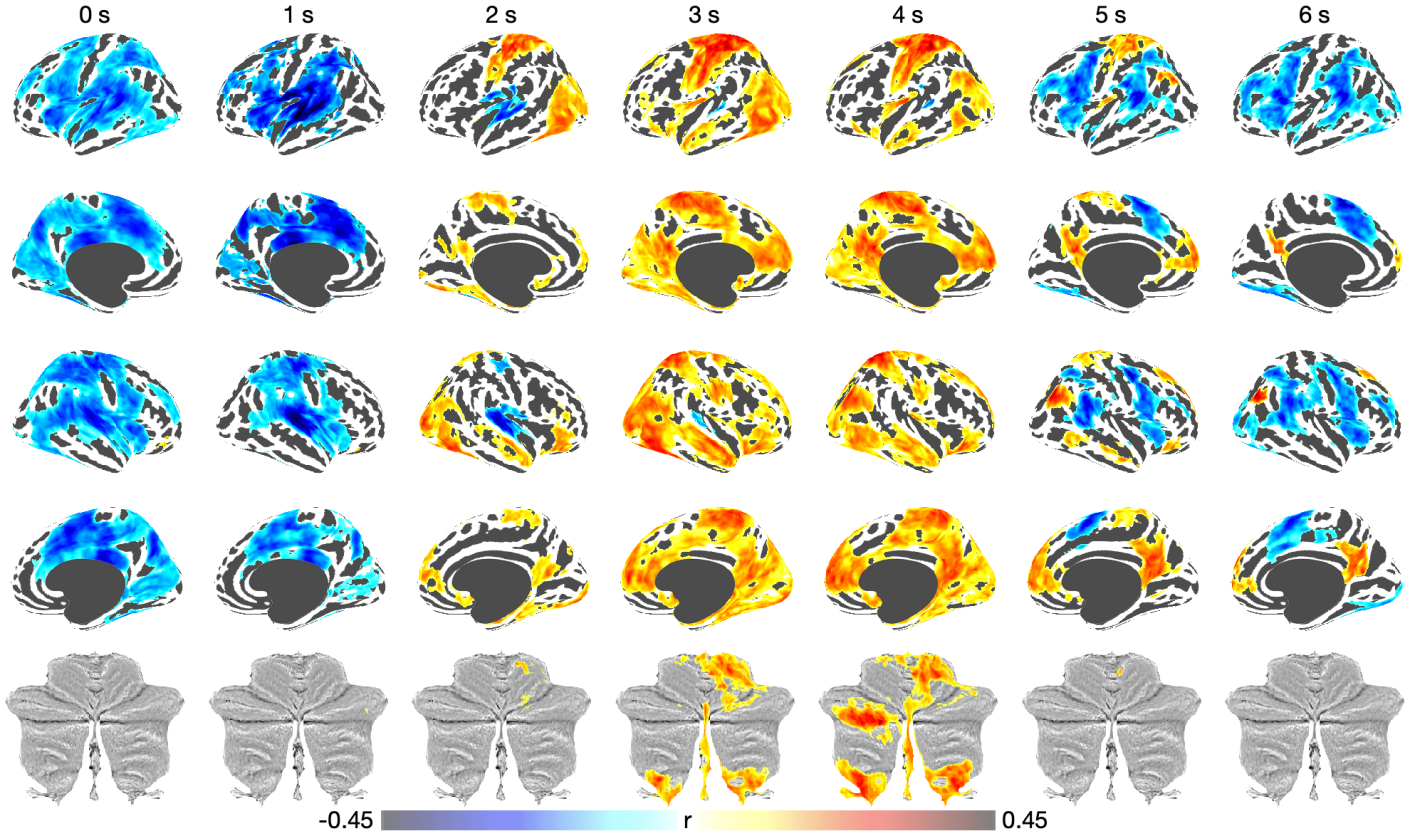
Brain–behavior associations during finger tapping. Voxel-wise correlations were computed between the absolute deviation of the inter-tap interval (ITI) from the 500-ms target interval and the parameter estimates obtained from the FIR analysis, accounting for the hemodynamic response function (HRF) delay from 0 to 6 sec. Colors indicate significant associations at TFCE-corrected *p* < 0.05.

We next tested whether the participant’s tapping behaviors (i.e., ITI and its CV) were associated with the functional activation during the entire tone-paced and self-paced tapping. The finite impulse response (FIR) model in FSL was used to extract the functional activation at each tone onset, assuming a 0.5 sec inter-tone interval continued during self-paced (i.e., continuation) tapping, which considered the delay of hemodynamic response function (HRF) from 0 to 6 sec. The ITI was converted to represent the deviation from the 0.5 sec of inter-tone interval. In Figure 5, we illustrated the voxel-wise correlations across participants between the absolute deviation of the inter-tap interval (ITI) from the 500-ms target interval and the time-resolved fMRI parameter estimates obtained from the finite impulse response (FIR) analysis. By modeling the hemodynamic response function (HRF) over a 0–6 sec delay window, this analysis captured dynamic changes of brain–behavior coupling in distinct brain regions across time (TFCE-corrected *p* < 0.05). Specifically, significant negative correlations were observed in the precentral, supplementary motor, inferior frontal, medial frontal, superior temporal, superior parietal, middle cingulate, and insula areas for the short delay (0-1 sec), positive correlations in the postcentral, paracentral, medial prefrontal, occipital, anterior/posterior cingulate, and cerebellum (lobule I-IV and VIIIa/b) for the middle delay (2–4 sec), and distributed negative and positive correlations for the large delay (5–6 sec) including the posterior cingulate, medial frontal regions (positive correlations) and the precentral, pre-supplementary motor, inferior parietal, inferior frontal, and middle frontal regions (negative correlations), respectively. These regions showed the associations between greater ITI deviation (i.e., worse temporal precision) and increased (positive correlation) and decreased (negative correlation) BOLD responses during both synchronization and continuation phases, which also revealed that different brain regions exhibited distinct temporal dynamics. The spatial distribution of these effects suggests that participants who exhibited greater variability in finger-tapping timing engaged auditory–motor integration networks more strongly to maintain temporal synchronization. Supplementary results of CV_ITI_ (which was less dynamic compared to ITI) and tapping force are represented in Supplementary Figure S5.

### 3.7 Comparison between SMA and event-based averaging methods

Another approach to analyze inter-tap interval (ITI) and coefficient of variation (CV) in sensorimotor synchronization tasks involves tap-based averaging—that is, averaging the ITI or CV at each tap index (e.g., the n-th tap) across all participants (Repp, 2003). This traditional method is appropriate for identifying group-level trends at specific tap positions, provides temporal alignment for comparing experimental conditions (e.g., tone-paced vs. self-paced), and allows for simpler statistical comparisons than time-based averaging methods. It is most appropriate when the research goal is to examine static, sequence-based group patterns or average changes across the tap sequence. However, it inherently disregards precise time information, as the same tap index may occur at different time points across individuals who tap at different rates. Consequently, this approach can be more sensitive to inter-individual variability, especially when the number of taps is limited or task duration is extended (e.g., a 20-sec continuation phase, as in this study). To address these limitations and better capture dynamic performance over time, we decided to use a sliding-window moving averaging (SMA) method. This approach averages ITI and CV values within a ±1-sec window around each time point (window size evaluated in Supplementary Fig. S3), enabling a temporally grounded and smoothed representation of behavioral fluctuations. As shown in Supplementary Figure S6, the SMA method reproduced the same distinctions across phase boundaries (early and late synchronization, early and late continuation) as the n-th tap averaging method for ITI. While CV showed more pronounced variability over time—especially during continuation—the SMA method provided a moderate smoothing effect, reducing inter-individual variability and noise while preserving the temporal structure of the data without distortion. Descriptive statistics yielded largely identical estimates of ITI and CV across the task phases for both methods. Crucially, when brain–behavior correlations were re-analyzed using ITI values derived from the traditional n-th tap method (Supplementary Fig. S6), all key patterns reported in Section 3 were preserved. This indicates that our SMA approach did not distort or inflate the main findings and, in fact, provided results consistent with conventional event-based averaging. Therefore, the SMA method not only retains the robustness of traditional analyses but also offers the added benefit of tracking temporal variability and rhythmic stability as they evolve during the task. This makes it particularly well-suited for time-based investigations, such as monitoring real-time rhythm maintenance, fatigue, attentional shifts, or the dynamic performance profiles of individual participants.

## 4 Discussion

This study examined distinct neural patterns during a sensorimotor synchronization task involving externally-cued, tone-paced tapping (synchronization) and internally-cued self-paced tapping (continuation). Four main findings emerged. First, tapping performance differed between early and late phases in both tapping conditions: accuracy increased and variability decreased rapidly within a few seconds during early tone-paced tapping, stabilizing in the late phase, while early self-paced tapping showed similar higher fluctuations before also settling in the late phase. Second, fMRI revealed higher activation in the early phases compared to the late phases, with distinct activation patterns in sensorimotor and related networks. Third, self-paced tapping engaged broader networks, including the prefrontal-parietal-temporal and salience networks, compared to tone-paced tapping. Fourth, brain-behavior correlations indicated that higher tapping accuracy and lower variability were associated with stronger activations in sensorimotor, prefrontal-parietal-temporal, salience networks, and the cerebellum. These findings support hypotheses that: (i) tapping accuracy and variability rapidly stabilizes as an internal timing model forms; (ii) functional brain involvement decreases as this internal model established; (iii) transitioning to self-paced tapping engages prefrontal-parietal-temporal networks more strongly; and (iv) sensorimotor synchronization performance correlates with functional activation in key brain regions.

### 4.1 Temporal dynamics of tapping behavior

Optimal sensorimotor synchronization relies on the brain’s ability to generate internal cues and correct for desired motor outputs through error correction (Sohn et al., 2021). Error detection and correction are essential to prevent the accumulation of motor variability (Repp, 2005). Internal feedback signals are compared to external cues to compute prediction errors, requiring an internal model to predict sensory inputs. As the model is refined, prediction errors decrease, supporting coordination for optimal behavior. During continuation, the absence of external cues may increase prediction errors, but the internal model adapts using memory of past errors to maintain motor output (Herzfeld et al., 2014). The adapted internal model, which might not be the same as the initial model, subsequently *maintains* the motor output without external cues. As expected, distinct behavioral patterns emerged between externally-cued tone-paced (synchronization) and internally-cued self-paced (continuation) tapping (Fig. 2). During early tone-paced tapping (Sync1), the inter-tap interval (ITI) increased before stabilizing in the late phase (Sync2), while variability decreased. This phase likely reflects the *tuning-in* stage required to initialize an internal model for coordinating taps with external tones, taking approximately 2.5 sec (4 finger taps), consistent with prior findings (Fraisse, 1966; Repp, 1999; Semjen et al., 1998). The finding is comparable to the previously known 2.4 sec (i.e., 3 taps with an inter-onset interval of 800-ms) when tapping starts immediately after the first tone, while the duration needed to reach the steady state is a function of the inter-onset interval (Fraisse, 1966). Participants initially tapped faster, likely influenced by the natural motor period of ~250 ms (Roberts et al., 2000). Once tones were discontinued, requiring self-paced tapping (Cont1), ITI and variability increased sharply (~1 sec), presumably due to heightened prediction errors from the absence of external cues. However, using memory of prior prediction errors, tapping performance quickly stabilized (Cont2), though ITI during this phase remained longer than in Sync2, indicating a deviation in the modified internal model. In the late self-paced phase, variability increased over time, possibly due to fatigue or reduced attention (Repp, 2005).

### 4.2 Functional activation of synchronization and continuation

Previous studies on sensorimotor synchronization highlight key regions, including the primary motor and premotor cortices, SMA, prefrontal and parietal cortices, basal ganglia, and anterior cerebellum (Turesky et al., 2018; von Schnehen et al., 2022; Witt et al., 2008). During early tone-paced tapping (Sync1), we observed activation in the primary/supplementary motor areas, anterior and posterior cerebellum (lobules V/VI, VIIIa/b), and ventral lateral thalamus (Fig. 3, Supplementary Table S1), consistent with our previous finger tapping study (Moussa-Tooks et al., 2019) and reflecting the cortico-cerebellar-thalamo-cortical circuit’s role (Ito, 1970; Strick et al., 2009; Tanaka et al., 2020) in motor planning (Gao et al., 2018). The dorsolateral prefrontal, anterior parietal, and superior temporal cortices were also active, suggesting involvement of the prefrontal-parietal-temporal network in temporal coordination, sensory (tone) – motor (tap) integration (Repp & Su, 2013), and attentional control (Guterstam et al., 2021; Noel et al., 2024). Additionally, the salience network regions (insula and middle cingulate) were active, presumably aiding in error monitoring and motor correction (Menon & Uddin, 2010). Crucial roles of the salience network include detecting behaviorally relevant stimuli (e.g., directing attention to tones), processing the critical aspects of stimuli (e.g., temporal rhythm of tones), coordinating motor responses (e.g., finger taps) in a synchronized manner, and initiating correction of discrepancies between the expected and actual outcomes (e.g., timing errors in tapping) by self- and error-monitoring (Menon & Uddin, 2010). In contrast, the late phase of the tone-paced condition showed minimal activation, limited to contralateral motor cortices and ipsilateral anterior cerebellum, indicating reduced network involvement as the internal model was established early through rapid adaptation (Wolpert & Flanagan, 2016). The distinct behavioral and neural patterns observed in early versus late synchronization may reflect refinement of internal model predictions rather than complete adaptation of the internal model, given the stable effector and environmental conditions. Transient neural and behavioral differences observed in early versus late synchronization phases could reflect the dynamic allocation of neural resources or shifts in attentional and motor control processes, rather than repeated adaptations of the internal model. These transient patterns may facilitate recalibration or optimization of synchronization mechanisms, even in the absence of changes to the effector or external world. These changes could represent the brain’s strategy for fine-tuning synchronization and minimizing asynchrony over trials. This process might involve fluctuations in attention, error correction, or neural coordination patterns without necessitating fundamental changes to the internal model itself. Self-paced (continuation) tapping activated shared regions in both early (Cont1) and late (Cont2) phases, including the primary motor, premotor, SMA, inferior frontal cortex, insula, middle cingulate, and basal ganglia (Fig. 3, Supplementary Table S2), consistent with prior meta-analyses (Witt et al., 2008). Greater activation in the early phase was observed in regions such as the right inferior frontal, superior/inferior parietal, middle temporal cortices, insula, middle cingulate cortex, and thalamus. Temporal cortex activation shifted from the middle region in the early phase to the superior region in the late phase. Cerebellar activation was more distinct in the late phase and limited to vermal regions. These findings suggest that early self-paced tapping involves greater cortical engagement for rapid internal model revision, while late-phase tapping relies more on the cerebellum—presumably for employing the revised model.

### 4.3 Comparison of brain activation during each timing phase

Brain activation during early self-paced tapping (Cont1) differed significantly from early tone-paced tapping (Sync1; Fig. 4, Supplementary Table S3). In the early tone-paced phase, higher activation was observed in the bilateral superior temporal cortex and contralateral cerebellum, likely reflecting the processing of external auditory cues (Janata & Grafton, 2003; Lewis et al., 2004). Unexpectedly, greater contralateral activation in motor (VI) and cognitive (Crus I) cerebellar lobules during tone-paced tapping may indicate differentiated cortico-cerebellar connectivity in the right cortical hemisphere. The underlying mechanism is not known, but it could be explained by the notion that the ipsilateral cortical regions more strongly modulate the contralateral cerebellum than the extent to which the contralateral cortical regions do. Conversely, early self-paced tapping showed greater activation in the inferior frontal, superior parietal, middle temporal, medial frontal, occipital regions, and right anterior insula, areas linked to executive control and multisensory processing (Harry et al., 2023). These findings suggest a critical role of the prefrontal-parietal-temporal network in reconstructing the internal model during the transition to self-paced tapping, with increased working memory demands for temporal representation in the absence of external cues (Jantzen et al., 2007).

Activation during late self-paced tapping (Cont2) was higher than during late tone-paced tapping (Sync2; Fig. 4, Supplementary Table S3). Regions showing increased activation included the sensorimotor cortex, SMA, insula, middle cingulate (including the rostral part; Jocham et al., 2009), middle temporal and inferior parietal cortices, basal ganglia (including amygdala), and cerebellum (lobules V-VI and VIIIa/b). These areas, part of the sensorimotor and salience networks, likely reflect the use of a revised internal model with ongoing self-monitoring to maintain rhythm (Harry et al., 2023; Koziol et al., 2014; Lewis et al., 2004). This finding aligns with prior studies (Habas et al., 2009; Witt et al., 2008) and highlights the increased functional demand for employing the reconstructed internal model during self-paced tapping. Compared to late synchronization (Sync2), late continuation (Cont2) was associated with greater involvement of regions such as the ventral motor area, SMA, insula, and cerebellum, as well as additional contributions from the middle cingulate, middle temporal, and inferior parietal cortices. These activations suggest enhanced self-monitoring and repeated recall of the rhythm to ensure alignment with the target pace in the absence of external cues.

### 4.4 Brain-behavior correlation

Distinct patterns of brain–behavior relationships were identified during the synchronization (tone-paced) and continuation (self-paced) phases. ITI deviations from the 500-ms inter-stimulus interval were associated with the magnitude of BOLD activity in regions engaged in the sensorimotor task. In addition to the somatomotor network (precentral, postcentral, and supplementary motor regions), significant associations with tapping performance were observed in prefrontal–parietal–temporal regions, including the inferior and middle frontal, medial frontal, and inferior parietal cortices—areas also identified in the fMRI activation analysis (Fig. 5). Regions showing negative correlations between BOLD activity and tapping error suggest effective recruitment of task-relevant neural circuits that support precise sensorimotor coordination. Stronger activation in prefrontal–parietal–temporal, auditory, and motor cortices may reflect enhanced sensorimotor coupling and efficient integration of external auditory cues with internal motor timing, enabling more stable tapping performance. These relationships are consistent with previous findings showing that greater engagement of the auditory–motor network—including the superior temporal gyrus, supplementary motor area, and cerebellum—facilitates accurate temporal prediction and synchronization, thereby improving performance (Anwar et al., 2016; Lewis et al., 2004; Luft et al., 2020). Conversely, regions showing positive correlations between BOLD activity and tapping error likely reflect inefficient or compensatory neural recruitment during temporal synchronization. Greater activation in these areas may indicate that participants with poorer temporal precision required increased neural effort to maintain task performance, suggesting reduced processing efficiency within sensorimotor and auditory–motor integration networks. Heightened activity in premotor, supplementary motor, and cerebellar regions may thus represent compensatory engagement to counteract variability in timing control, consistent with the notion that individuals with less stable synchronization rely on broader or more intensive neural resources to achieve comparable outcomes. Overall, these distinct patterns may reflect the differential roles of cortical and subcortical regions in error monitoring and the construction, maintenance, and correction of internal timing models during synchronization and continuation. In contrast, the coefficient of variation of ITI did not reveal a distinct regional pattern compared to ITI itself (Supplementary Fig. S5), suggesting that this measure may be less directly related to the brain–behavior relationship observed in this task. Notably, in our FIR model, each β coefficient represented the BOLD response at a specific time lag following stimulus onset. Correlations with behavioral measures were computed for each time point, capturing the temporal dynamics of the neural response as shown in Figure 5. This task-based approach allowed us to assess trial-by-trial brain–behavior relationships by directly linking participants’ tapping performance to their corresponding brain activity. While this method effectively identified brain regions functionally associated with behavior, the actual delay of the hemodynamic response likely varied across regions (Supplementary Fig. S7) and was not predefined in this model. Therefore, interpretations regarding the exact timing of activation onset or peak should be made cautiously. Rather, our findings emphasize distinct spatial patterns of brain regions associated with tapping performance across time.

### 4.5 Limitations

This study has several limitations. First, our results from the early and lates phases of the synchronization-continuation task were generally consistent with Gentile’s two-stage model of motor learning, comprising the fixation and diversification stages (Gentile, 1972). Because the external rhythm in our finger-tapping synchronization task provided a consistent, closed environmental stimulus, the observed sensorimotor learning can be primarily interpreted within Gentile’s *fixation* stage, in which the performer refines a stable movement pattern under constant conditions. However, since the task involved little environmental variability, the *diversification* aspect of Gentile’s model is likely less applicable. Therefore, three-stage motor learning models—such as the cognitive, associative, and autonomous stages (Fitts & Posner, 1967) and the freezing, releasing, and exploiting stages (Bernstein, 1996)—may provide a more appropriate framework to describe the progressive improvement in timing accuracy observed in this task. Nonetheless, our tapping results did not clearly align with all three phases, possibly due to the task’s simplicity and short adaptation period, during which the associative process may have overlapped. More complex paradigms may be better suited to validate these stage transitions in the future study. Second, synchronization performance (Repp, 2005; Witt et al., 2008) and neural networks (von Schnehen et al., 2022) vary with the modality and rate of external cues. Using a fixed 500-ms auditory interval in this study limits generalizability, as tapping behavior may vary with cue complexity or inter-onset intervals. Future studies should explore broader intervals and complexity to assess parametric effects like previous studies (Flach, 2005; Lewis et al., 2004; Repp & Keller, 2008). Third, variability within timing phases remains unclear. Though tapping variance can reflect independent contributions of the internal clock and motor activity (Wing & Kristofferson, 1973), our short early (2.5 sec) and late (3.5 sec) tone-paced tapping durations may reflect fast internal model processing (Smith et al., 2006). Further research is needed to determine if tapping variability depends on timing phases. Finally, fMRI noise may have influenced the behavioral task, as its regular (periodic) component could act as a distracting auditory stimulus, potentially affecting participants’ performance (Haller & Bartsch, 2009). In our study, the fMRI scanner operated with a repetition time (TR) of 1.0 sec. Since the TR was a multiple of the designated inter-onset interval (IOI) of 0.5 sec, overlapping stimulus and scanner noise patterns may have interfered with participants’ tapping performance.

### 4.6 Conclusion

Our analysis revealed distinct behavioral and neural patterns in sensorimotor synchronization and continuation based on early versus late phases of task conditions (tone-paced vs. self-paced). In both conditions, early phases showed abrupt changes in inter-tap intervals and variability. Subsequent research will need to test specific hypotheses about these brain-behavior relationships.

## Supplementary Material

Supplementary Material

## Data Availability

The code will be made available upon request. The data cannot be shared publicly due to ethics approval restrictions and can be shared only with explicit approval from the research ethics board. Data and code can be accessed by contacting D.J.K. and W.P.H.
