# Supplementary material for: Distinct neural signatures in a sensorimotor synchronization-continuation task
Source: Imaging Neurosci (Camb). 2026 Jan 14;4:IMAG.a.1100. doi: 10.1162/IMAG.a.1100 (PMC12809721; doi:10.1162/IMAG.a.1100)
Supplement: Supplementary Material [file IMAG.a.1100_supp.pdf]

Supplementary materials for

Distinct neural signatures in a sensorimotor synchronization-continuation task

Dae-Jin Kim[1,*], Amanda R. Bolbecker[1], Alexandra B. Moussa-Tooks[1,2,3], Krista M. Wisner[1,2], Brian F. O'Donnell[1,2], Emily L. Gildea[1], William P. Hetrick[1,2,3]

[1] Department of Psychological & Brain Sciences, Indiana University, Bloomington, IN, USA

[2] Program of Neuroscience, Indiana University, Bloomington, IN, USA

[3] Department of Psychiatry, Indiana University School of Medicine, Indianapolis, IN, USA

* Corresponding author

Dae-Jin Kim Ph.D.

E-mail: daejkim@iu.edu

Department of Psychological and Brain Sciences

Indiana University

1101 E 10th Street, Bloomington, IN 47405

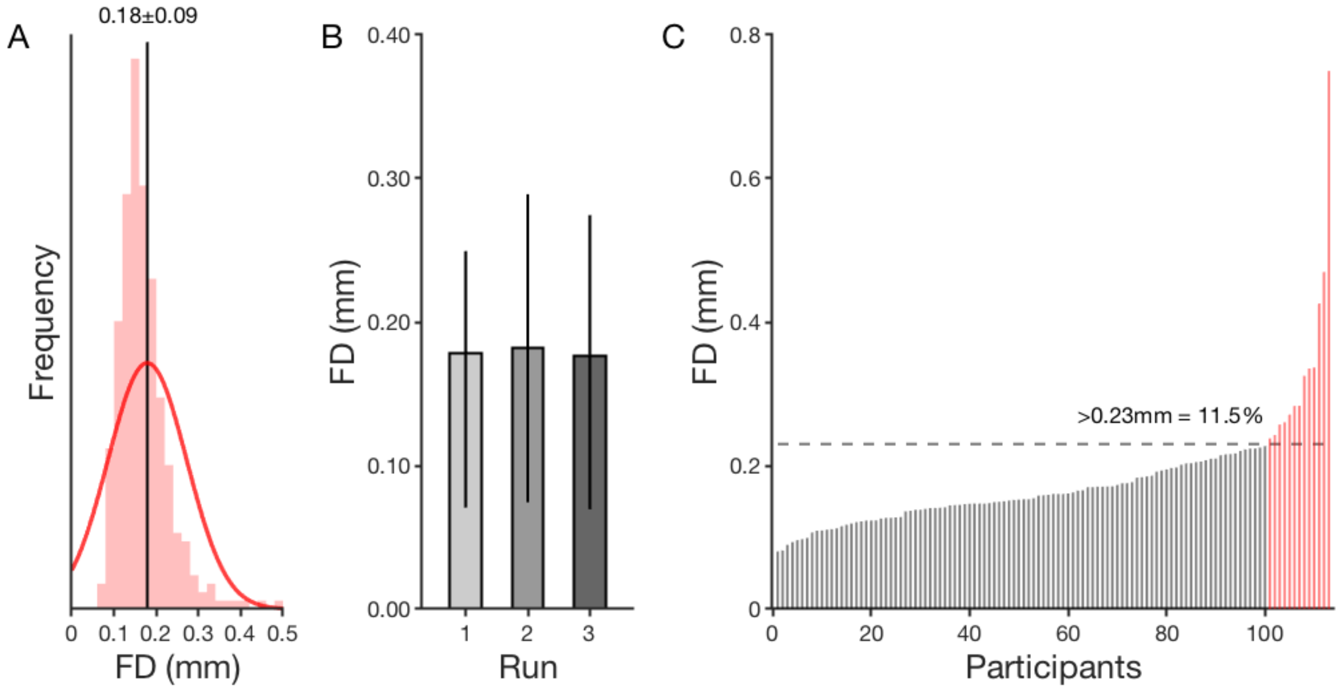

Fig S1. Analysis of head motion. The head movement during fMRI scanning was assessed using framewise displacement (FD), which quantifies the root mean squared head motion change between successive scans (Power et al., 2012). **A**. The histogram of FD averaged across runs showed a mean and standard deviation of 0.18 ± 0.09 mm (black vertical line) with an approximated Gaussian curve (red). **B**. A one-way ANOVA revealed no significant differences in head movement across runs, $F(2,336) = 0.08$, $DF = 2$, $P = 0.919$, $\eta^2 = 0.0005$. **C**. To minimize motion-related artifacts in the fMRI analysis, three strategies were implemented: (i) participants with FD values exceeding 0.23 mm (a less conservative threshold chosen due to increased task motion compared to resting-state scans) were excluded, resulting in the removal of 13 participants (11.5%, highlighted in red); (ii) ICA-based motion confound regression (Pruim et al., 2015) was applied during preprocessing to reduce motion-correlated noise in the BOLD signal; and (iii) FD values were included as covariates in the general linear model (GLM) analysis, including FIR analysis, to account for residual motion effects. These measures ensured robust control of motion-related noise and improved the reliability of the fMRI results.

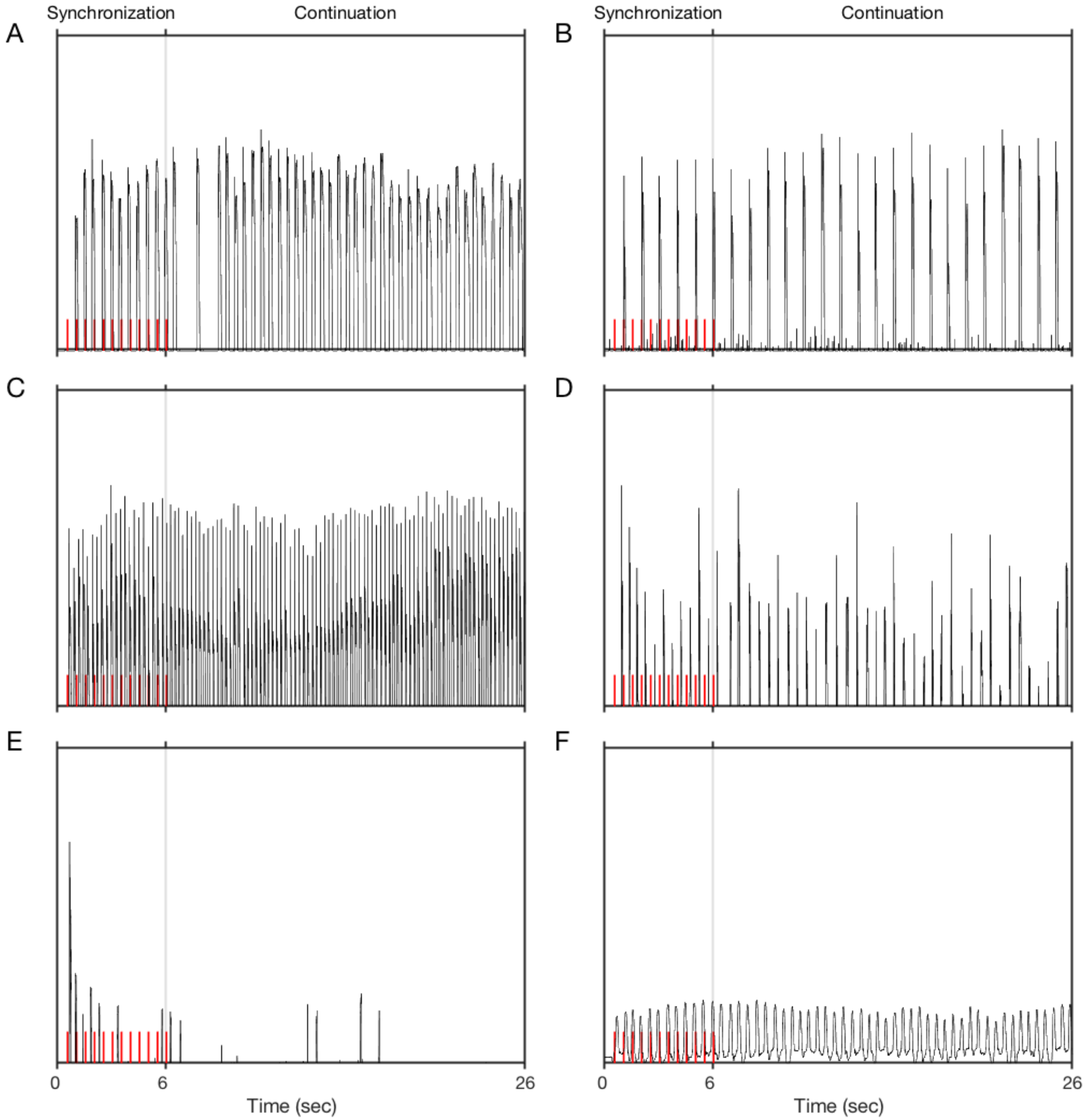

Fig S2. Patterns of excluded finger-tapping signals. A. Inter-tap intervals (ITIs) were excluded from the analysis if they exceeded 1000 ms, as this indicates a missed tap. **B-F.** Examples of excluded tapping patterns include slow tapping (**B;** ITI >1000 ms), fast tapping (**C**; ITI <250 ms), weak tapping (**D**; Force <500 a.u.), sparse tapping (**E**; fewer than 20 measured peaks per block), and irregular tapping signals (**F**). Red bars in the synchronization indicate tone triggers.

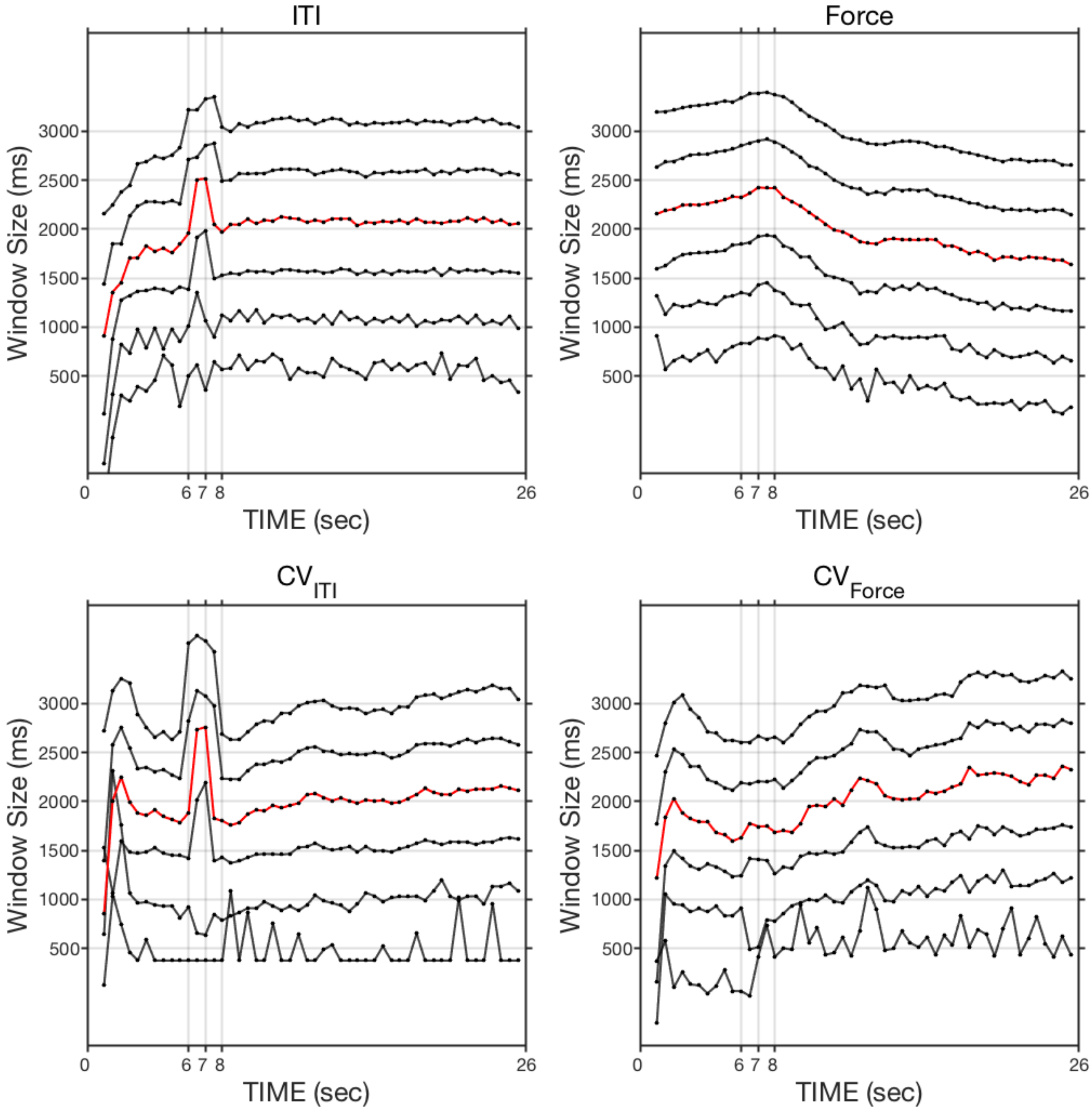

Fig S3. Size effect of sliding-window on computing dynamics of tapping behaviors. Each measure was computed using a sliding-window moving average (SMA) method every 500 ms. While the temporal dynamics showed similar patterns regardless of the window size, the smaller window size (500-1000 ms) showed more fluctuated patterns of temporal dynamics and the larger window size (2500-3000 ms) indicated a smoother effect. The red line represents the results with a window size of 2000 ms in the main manuscript.

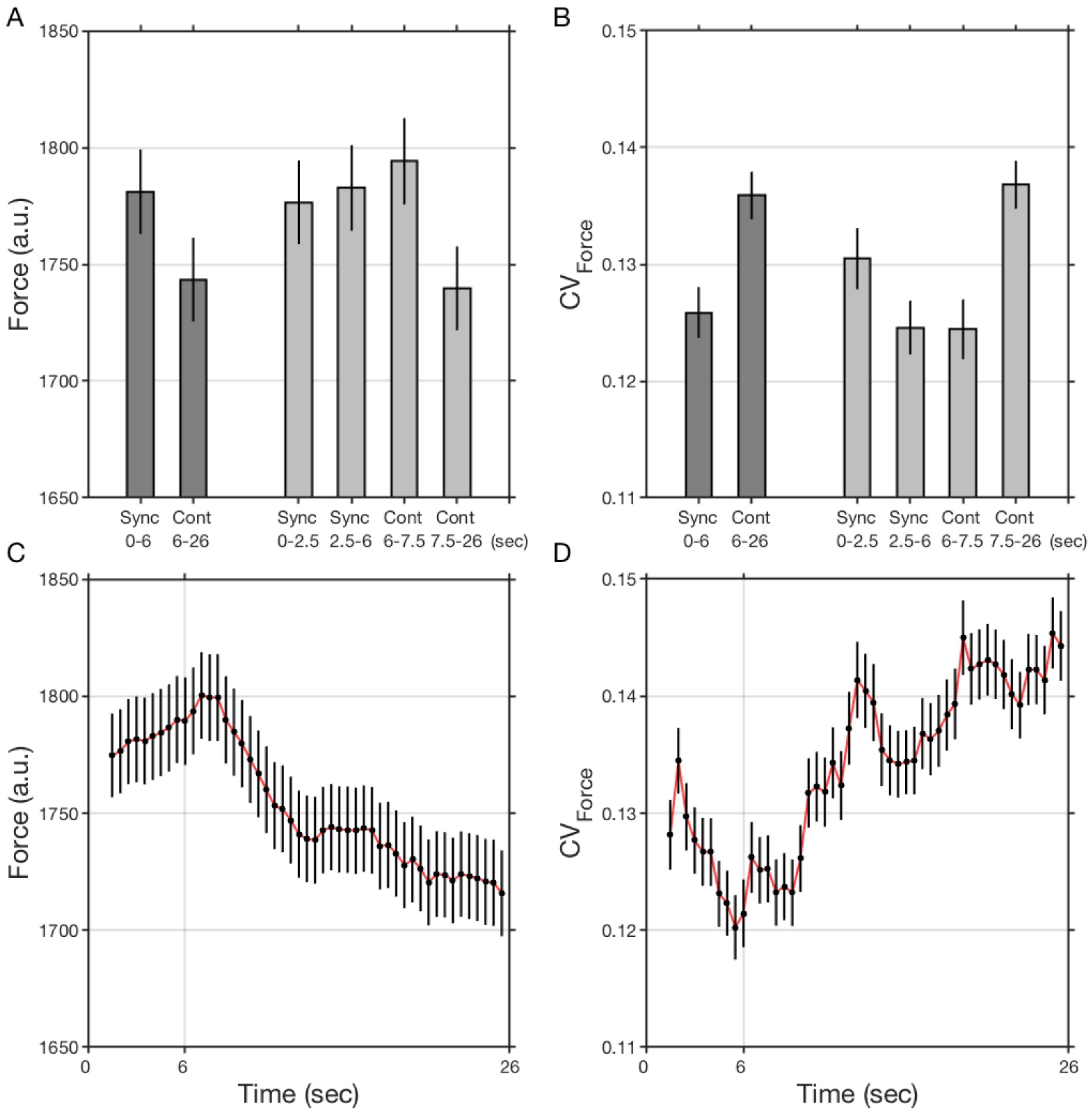

Fig S4. Analysis of tapping force. (A) Magnitude of tapping force and **(B)** the coefficient of variation (CV) of force. Temporal dynamics of **(C)** tapping force and **(D)** CV. Compared to inter-tap interval (ITI), less distinct patterns of tapping characteristics were observed in force (**C**) and its CV (**D**). Error bars (black) represent the standard error of the mean.

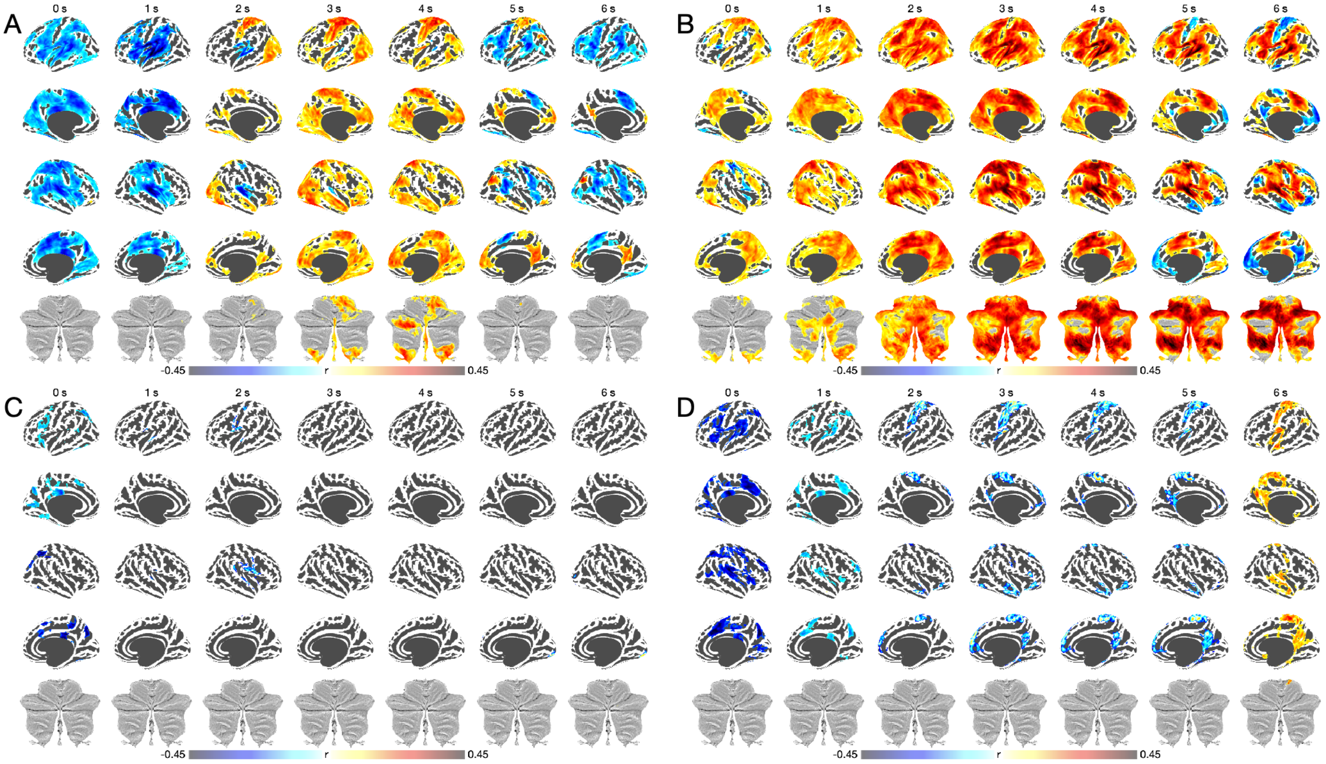

Fig S5. Brain-behavior association of tapping measures. Voxel-wise correlations were computed between the parameter estimates obtained from the FIR analysis, accounting for the hemodynamic response function (HRF) delay from 0 to 6 s, and (**A**) the absolute deviation of the inter-tap interval (ITI) from the 500-ms target interval, (**B**) tapping force, and (**C-D**) their coefficients of variation (CV). Colors indicate significant associations at TFCE-corrected $p < 0.05$.

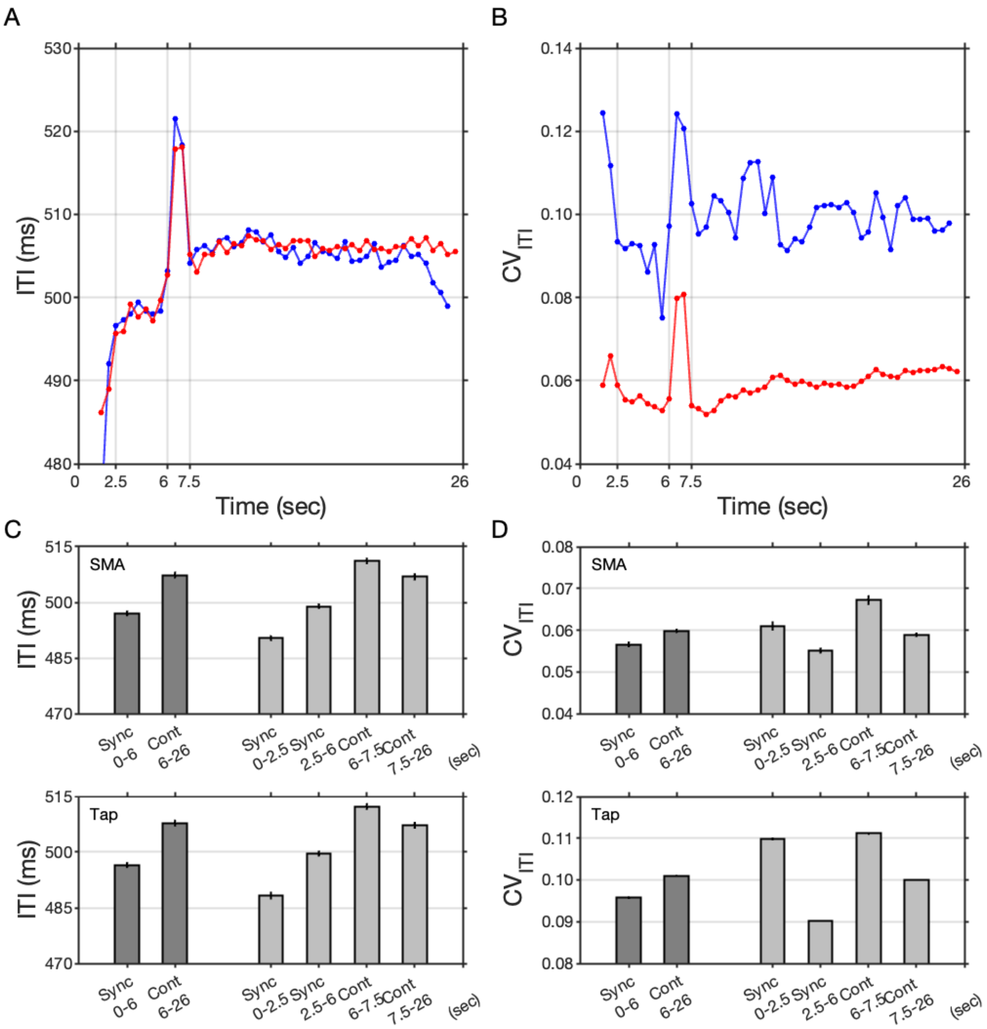

Fig S6. Comparison between SMA and tap-based averaging methods. ITI (**A**) and CV (**B**) for SMA (red) and n-th tap averaging method (blue). **C-D.** Descriptive statistics for each condition (dark gray) and phase (light gray) for SMA (top) and n-th tap averaging (bottom).

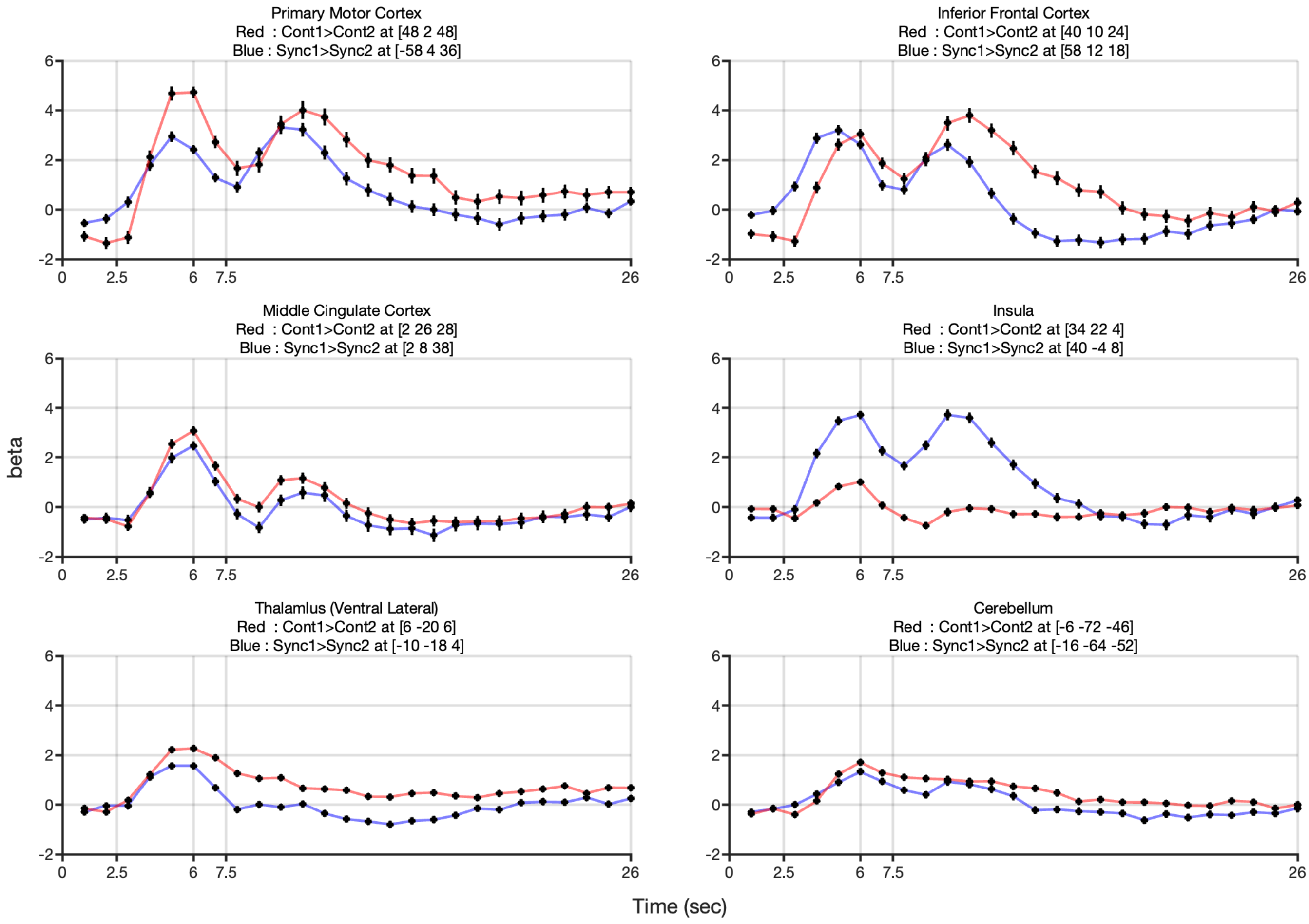

Fig S7. Examples of the parameter estimates extracted from FIR analysis. The group-averaged β-weights for the selected regions corresponding to some significant results reported in Tables S1–S3 were displayed.

References

Power, J. D., Barnes, K. A., Snyder, A. Z., Schlaggar, B. L., & Petersen, S. E. (2012). Spurious but systematic correlations in functional connectivity MRI networks arise from subject motion. *Neuroimage*, *59*(3), 2142–2154. https://doi.org/10.1016/j.neuroimage.2011.10.018

Pruim, R. H. R., Mennes, M., van Rooij, D., Llera, A., Buitelaar, J. K., & Beckmann, C. F. (2015). ICA-AROMA: A robust ICA-based strategy for removing motion artifacts from fMRI data. *Neuroimage*, *112*, 267–277. https://doi.org/10.1016/j.neuroimage.2015.02.064

Table S1. Peaks of functional activation of early and late synchronization.

Anatomical region		Sync1 > Rest				Sync2 > Rest				Sync1 > Sync2				Sync2 > Sync1			
		***x*[a]**	***y***	***z***	***Z***	***x***	***y***	***z***	***Z***	***x***	***y***	***z***	***Z***	***x***	***y***	***z***	***Z***
Motor	Primary (M1)	-44	-18	56	8.98	-40	-20	48	9.23	-58	4	36	8.47				
	Supplementary	0	-2	57	9.34	-6	-6	60	6.00	0	0	56	8.77				
	Somatosensory	52	-42	48	7.66					52	-42	48	6.47				
		-58	-22	42	9.60					-58	-22	40	9.01				
Temporal	Superior	58	-26	12	9.70					58	-10	2	9.92				
		-58	-20	6	10.61					-56	-18	6	10.61				
Frontal	Inferior	54	10	10	8.50					58	12	18	7.69				
		-58	10	14	7.71					-58	10	14	7.53				
	Middle	40	44	30	7.18	36	48	32	5.55	36	42	36	6.28				
		-42	42	26	7.40					-44	42	22	6.92				
Insula		42	0	8	5.94					40	0	10	7.61				
		-40	-2	8	8.11					-40	-4	8	8.82				
Cingulate	Middle	4	8	38	8.09					2	8	38	9.11				
		-6	8	34	7.84					-6	8	36	8.00				
	Posterior	0	-24	44	6.87					0	-24	44	8.80				
Occipital	Lateral	10	-74	34	6.30					10	-74	32	7.35				
		-10	-72	34	7.07					-8	-74	32	7.05				
Subcortex	Putamen	20	4	6	5.95					26	-2	6	4.57				
		-26	-2	6	6.07					-28	-12	4	4.92				
	Thalamus (Ventral Lateral)	14	-18	4	4.46					12	-18	4	5.14				
		-10	-18	2	5.64					-10	-18	4	5.50				
	Amygdala	24	2	-14	5.33					24	0	-16	7.27				
		-24	0	-14	5.50					-24	0	-16	7.17				
Cerebellum	V	2	-58	-8	7.72	18	-48	-28	5.50	2	-58	-8	6.82				
	VI	24	-56	-26	9.48	24	-54	-26	5.25	22	-66	-24	8.16				
		-28	-66	-26	9.20					-22	-61	-26	8.76				
	VIIIa/b	14	-60	-52	8.97					14	-60	-52	8.72				
		-18	-64	-54	7.79					-16	-64	-52	8.18				
	VIIIa (Vermis)	4	-67	-36	6.98					0	-70	-36	7.44				

Note. Clusters in each group were thresholded at $Z>3.1$ and corrected for multiple comparisons in the whole brain ($P<0.05$). No functional activation was found for Sync2>Sync1. MNI coordinates (*x*, *y*, and *z*) were used in mm, in which positive and negative values in *x* represent the right and left hemispheres, respectively. Anatomical regions were assigned at peak voxels within clusters using Harvard–Oxford Cortical and Subcortical Probabilistic Structural Atlases, SUIT Cerebellar Atlas, and Oxford Striatal Connectivity Atlas.

Table S2. Peaks of functional activation of early and late continuation.

Anatomical region		Cont1 > Rest				Cont2 > Rest				Cont1 > Cont2				Cont2 > Cont1			
		x^a	*y*	*z*	*Z*	*x*	*y*	*z*	*Z*	*x*	*y*	*z*	*Z*	*x*	*y*	*z*	*Z*
Motor	Primary (M1)	48	2	48	8.20	56	2	40	9.25	48	2	48	7.62				
		-48	-2	50	7.82	-44	-18	46	11.47	-48	-2	50	7.12				
	Supplementary	2	6	56	7.89	6	-2	64	10.96	4	6	54	7.19				
						-4	-4	58	11.07	0	6	50	7.05				
	Somatosensory	52	-42	10	9.60	54	-40	52	7.80	52	-42	10	9.50				
		-54	-48	10	8.49	-58	-38	50	6.61	-54	-48	10	8.28				
Temporal	Superior					60	-32	18	8.82					58	-8	0	6.12
						-50	-38	18	10.68					-50	-10	0	5.96
	Middle	52	-28	-6	7.20					48	-24	-8	7.15				
		-56	-48	10	8.24												
Frontal	Inferior	42	10	24	8.68	40	10	24	8.69	40	10	24	8.50				
										-44	10	22	5.57				
	Middle					36	42	20	7.33								
						-36	36	20	6.70								
Insula		38	20	-2	7.01	40	8	-2	10.40	34	22	4	6.31				
		-40	20	-4	6.77	-36	4	2	9.82	-32	26	0	5.53				
Cingulate	Middle	4	26	28	6.49	6	12	40	8.93	2	26	28	6.29				
		-6	22	24	5.66	-12	4	34	8.82								
Occipital	Lateral	36	-88	-12	9.63	16	-88	-16	9.85	36	-88	-12	9.57				
		-42	-80	-14	9.33	-18	-88	-12	9.21	-32	-88	-14	9.02				
Subcortex	Putamen	-32	-8	-4	4.41	22	0	2	10.87								
						-26	-6	2	11.63								
	Thalamus (Ventral Lateral)	6	-20	6	6.04	8	-18	0	8.51	6	-20	6	5.86				
		-16	-20	10	5.42	-16	-20	4	11.34	-16	-18	12	5.17				
Cerebellum	V					10	-52	-18	11.01								
	VI					28	-60	-26	10.54								
						-30	-64	-26	9.06								
	VI (Vermis)	-2	-76	-22	5.60	4	-62	-26	10.23	-2	-76	-22	5.48				
	VIIIa/b	-6	-72	-46	5.27	12	-62	-50	9.57	-6	-72	-46	5.25				
						-26	-62	-55	8.02								
	X	14	-40	-46	3.98												

Note. Clusters in each group were thresholded at Z>3.1 and corrected for multiple comparisons in the whole brain (P<0.05). MNI coordinates in mm were used, with x, y, and z, where positive and negative values in *x* represent the right and left hemispheres, respectively. Anatomical regions were assigned at peak voxels within clusters using Harvard–Oxford Cortical and Subcortical Probabilistic Structural Atlases and SUIT Cerebellar Atlas.

Table S3. Peaks of functional activation of synchronization vs. continuation.

Anatomical region		Sync1 >Cont1				Cont1 > Sync1				Sync2 > Cont2				Cont2 > Sync2			
		x[a]	*y*	*z*	*Z*	*x*	*y*	*z*	*Z*	*x*	*y*	*z*	*Z*	*x*	*y*	*z*	*Z*
Motor	Primary (M1)													60	-10	36	7.06
														-54	-10	36	8.19
	Supplementary													-2	8	70	7.91
	Somatosensory					50	-40	10	6.76					58	-10	26	7.88
						-60	-52	12	5.18					-64	-10	24	8.72
Temporal	Superior	58	-8	0	7.53												
		-50	-10	0	7.51												
	Middle					46	-24	-10	5.18					46	-30	-4	6.50
						-50	-28	-10	4.09					-62	-28	-8	4.99
Frontal	Inferior					40	10	24	6.99								
						-44	10	24	3.85								
	Medial					6	14	50	4.11								
Insula														38	6	-14	7.68
														-38	6	-16	6.66
Parietal	Superior					-28	-54	46	5.17								
						32	-48	46	4.69								
	Inferior													52	-66	30	5.53
														-50	-66	32	6.41
Cingulate	Middle													0	6	36	8.27
	Posterior													-2	-26	44	8.11
Occipital	Lateral					36	-88	-12	8.80								
						-32	-88	-14	8.22								
	Medial													0	-60	28	5.67
Subcortex	Putamen													28	-14	2	4.71
														-30	-16	-2	4.99
	Thalamus (Ventral Lateral)													6	-20	6	6.92
														-10	-22	8	5.13
	Amygdala													22	-2	-18	7.86
														-24	2	-16	7.73
Cerebellum	VI	-26	-64	-26	4.46									18	-68	-28	3.89
														-22	-60	-24	5.23
	VI (Vermis)													0	-66	-36	5.01
	VIIIa/b													10	-62	-50	5.68
														-12	-60	-52	6.33
	Crus I	-52	-56	-34	4.06												

Note. Clusters in each group were thresholded at $Z>3.1$ and corrected for multiple comparisons in the whole brain ($P<0.05$). No functional activation was found for Sync2>Cont2. MNI coordinates in mm were used, with x, y, and z, where positive and negative values in x represent the right and left hemispheres, respectively. Anatomical regions were assigned at peak voxels within clusters using Harvard–Oxford Cortical and Subcortical Probabilistic Structural Atlases and SUIT Cerebellar Atlas.

fMRIPrep Boilerplate

Results included in this manuscript come from preprocessing performed using fMRIPrep 20.2.5 (Esteban, Markiewicz, et al. (2018); Esteban, Blair, et al. (2018); RRID:SCR_016216), which is based on Nipype 1.6.1 (Gorgolewski et al. (2011); Gorgolewski et al. (2018); RRID:SCR_002502).

Anatomical data preprocessing

A total of 1 T1-weighted (T1w) images were found within the input BIDS dataset.The T1-weighted (T1w) image was corrected for intensity non-uniformity (INU) with N4BiasFieldCorrection (Tustison et al. 2010), distributed with ANTs 2.3.3 (Avants et al. 2008, RRID:SCR_004757), and used as T1w-reference throughout the workflow. The T1w-reference was then skull-stripped with a Nipype implementation of the antsBrainExtraction.sh workflow (from ANTs), using OASIS30ANTs as target template. Brain tissue segmentation of cerebrospinal fluid (CSF), white-matter (WM) and gray-matter (GM) was performed on the brain-extracted T1w using fast (FSL 5.0.9, RRID:SCR_002823, Zhang, Brady, and Smith 2001). Brain surfaces were reconstructed using recon-all (FreeSurfer 6.0.1, RRID:SCR_001847, Dale, Fischl, and Sereno 1999), and the brain mask estimated previously was refined with a custom variation of the method to reconcile ANTs-derived and FreeSurfer-derived segmentations of the cortical gray-matter of Mindboggle (RRID:SCR_002438, Klein et al. 2017). Volume-based spatial normalization to two standard spaces (MNI152NLin2009cAsym, MNI152NLin6Asym) was performed through nonlinear registration with antsRegistration (ANTs 2.3.3), using brain-extracted versions of both T1w reference and the T1w template. The following templates were selected for spatial normalization: ICBM 152 Nonlinear Asymmetrical template version 2009c [Fonov et al. (2009), RRID:SCR_008796; TemplateFlow ID: MNI152NLin2009cAsym], FSL's MNI ICBM 152 non-linear 6th Generation Asymmetric Average Brain Stereotaxic Registration Model [Evans et al. (2012), RRID:SCR_002823; TemplateFlow ID: MNI152NLin6Asym],

Functional data preprocessing

For each of the 5 BOLD runs found per subject (across all tasks and sessions), the following preprocessing was performed. First, a reference volume and its skull-stripped version were generated by aligning and averaging 1 single-band references (SBRefs). Susceptibility distortion correction (SDC) was omitted. The BOLD reference was then co-registered to the T1w reference using bbregister (FreeSurfer) which implements boundary-based registration (Greve and Fischl 2009). Co-registration was configured with six degrees of freedom. Head-motion parameters with respect to the BOLD reference (transformation matrices, and six corresponding rotation and translation parameters) are estimated before any spatiotemporal filtering using mcflirt (FSL 5.0.9, Jenkinson et al. 2002). BOLD runs were slice-time corrected to 0.315s (0.5 of slice acquisition range 0s-0.63s) using 3dTshift from AFNI 20160207 (Cox and Hyde 1997, RRID:SCR_005927). First, a reference volume and its skull-stripped version were generated using a custom methodology of fMRIPrep. The BOLD time-series (including slice-timing correction when applied) were resampled onto their original, native space by applying the transforms to correct for head-motion. These resampled BOLD time-series will be referred to as preprocessed BOLD in original space, or just preprocessed BOLD. The BOLD time-series were resampled into standard space, generating a preprocessed BOLD run in MNI152NLin2009cAsym space. First, a reference volume and its skull-stripped version were generated using a custom methodology of fMRIPrep. Automatic removal of motion artifacts using independent component analysis (ICA-AROMA, Pruim et al. 2015) was performed on the preprocessed BOLD on MNI space time-series after removal of non-steady state volumes and spatial smoothing with an isotropic, Gaussian kernel of 6mm FWHM (full-width half-maximum). Corresponding “non-aggresively” denoised runs were produced after such smoothing. Additionally, the “aggressive” noise-regressors were collected and placed in the corresponding confounds file. Several confounding time-series were calculated based on the preprocessed BOLD: framewise displacement (FD), DVARS and three region-wise global signals. FD was computed using two formulations following Power (absolute sum of relative motions, Power et al. (2014)) and Jenkinson

(relative root mean square displacement between affines, Jenkinson et al. (2002)). FD and DVARS are calculated for each functional run, both using their implementations in Nipype (following the definitions by Power et al. 2014). The three global signals are extracted within the CSF, the WM, and the whole-brain masks. Additionally, a set of physiological regressors were extracted to allow for component-based noise correction (CompCor, Behzadi et al. 2007). Principal components are estimated after high-pass filtering the preprocessed BOLD time-series (using a discrete cosine filter with 128s cut-off) for the two CompCor variants: temporal (tCompCor) and anatomical (aCompCor). tCompCor components are then calculated from the top 2% variable voxels within the brain mask. For aCompCor, three probabilistic masks (CSF, WM and combined CSF+WM) are generated in anatomical space. The implementation differs from that of Behzadi et al. in that instead of eroding the masks by 2 pixels on BOLD space, the aCompCor masks are subtracted a mask of pixels that likely contain a volume fraction of GM. This mask is obtained by dilating a GM mask extracted from the FreeSurfer’s aseg segmentation, and it ensures components are not extracted from voxels containing a minimal fraction of GM. Finally, these masks are resampled into BOLD space and binarized by thresholding at 0.99 (as in the original implementation). Components are also calculated separately within the WM and CSF masks. For each CompCor decomposition, the k components with the largest singular values are retained, such that the retained components’ time series are sufficient to explain 50 percent of variance across the nuisance mask (CSF, WM, combined, or temporal). The remaining components are dropped from consideration. The head-motion estimates calculated in the correction step were also placed within the corresponding confounds file. The confound time series derived from head motion estimates and global signals were expanded with the inclusion of temporal derivatives and quadratic terms for each (Satterthwaite et al. 2013). Frames that exceeded a threshold of 0.5 mm FD or 1.5 standardised DVARS were annotated as motion outliers. All resamplings can be performed with a single interpolation step by composing all the pertinent transformations (i.e. head-motion transform matrices, susceptibility distortion correction when available, and co-registrations to anatomical and output spaces). Gridded (volumetric) resamplings were performed using

antsApplyTransforms (ANTs), configured with Lanczos interpolation to minimize the smoothing effects of other kernels (Lanczos 1964). Non-gridded (surface) resamplings were performed using mri_vol2surf (FreeSurfer). First, a reference volume and its skull-stripped version were generated using a custom methodology of fMRIPrep. Susceptibility distortion correction (SDC) was omitted. The BOLD reference was then co-registered to the T1w reference using bbregister (FreeSurfer) which implements boundary-based registration (Greve and Fischl 2009). Co-registration was configured with six degrees of freedom. Head-motion parameters with respect to the BOLD reference (transformation matrices, and six corresponding rotation and translation parameters) are estimated before any spatiotemporal filtering using mcflirt (FSL 5.0.9, Jenkinson et al. 2002). BOLD runs were slice-time corrected to 0.449s (0.5 of slice acquisition range 0s-0.897s) using 3dTshift from AFNI 20160207 (Cox and Hyde 1997, RRID:SCR_005927). The BOLD time-series (including slice-timing correction when applied) were resampled onto their original, native space by applying the transforms to correct for head-motion. These resampled BOLD time-series will be referred to as preprocessed BOLD in original space, or just preprocessed BOLD. The BOLD time-series were resampled into standard space, generating a preprocessed BOLD run in MNI152NLin2009cAsym space. First, a reference volume and its skull-stripped version were generated using a custom methodology of fMRIPrep. Automatic removal of motion artifacts using independent component analysis (ICA-AROMA, Pruim et al. 2015) was performed on the preprocessed BOLD on MNI space time-series after removal of non-steady state volumes and spatial smoothing with an isotropic, Gaussian kernel of 6mm FWHM (full-width half-maximum). Corresponding “non-aggresively” denoised runs were produced after such smoothing. Additionally, the “aggressive” noise-regressors were collected and placed in the corresponding confounds file. Several confounding time-series were calculated based on the preprocessed BOLD: framewise displacement (FD), DVARS and three region-wise global signals. FD was computed using two formulations following Power (absolute sum of relative motions, Power et al. (2014)) and Jenkinson (relative root mean square displacement between affines, Jenkinson et al. (2002)). FD and DVARS are calculated for each functional run, both using their

implementations in Nipype (following the definitions by Power et al. 2014). The three global signals are extracted within the CSF, the WM, and the whole-brain masks. Additionally, a set of physiological regressors were extracted to allow for component-based noise correction (CompCor, Behzadi et al. 2007). Principal components are estimated after high-pass filtering the preprocessed BOLD time-series (using a discrete cosine filter with 128s cut-off) for the two CompCor variants: temporal (tCompCor) and anatomical (aCompCor). tCompCor components are then calculated from the top 2% variable voxels within the brain mask. For aCompCor, three probabilistic masks (CSF, WM and combined CSF+WM) are generated in anatomical space. The implementation differs from that of Behzadi et al. in that instead of eroding the masks by 2 pixels on BOLD space, the aCompCor masks are subtracted a mask of pixels that likely contain a volume fraction of GM. This mask is obtained by dilating a GM mask extracted from the FreeSurfer's aseg segmentation, and it ensures components are not extracted from voxels containing a minimal fraction of GM. Finally, these masks are resampled into BOLD space and binarized by thresholding at 0.99 (as in the original implementation). Components are also calculated separately within the WM and CSF masks. For each CompCor decomposition, the k components with the largest singular values are retained, such that the retained components' time series are sufficient to explain 50 percent of variance across the nuisance mask (CSF, WM, combined, or temporal). The remaining components are dropped from consideration. The head-motion estimates calculated in the correction step were also placed within the corresponding confounds file. The confound time series derived from head motion estimates and global signals were expanded with the inclusion of temporal derivatives and quadratic terms for each (Satterthwaite et al. 2013). Frames that exceeded a threshold of 0.5 mm FD or 1.5 standardised DVARS were annotated as motion outliers. All resamplings can be performed with a single interpolation step by composing all the pertinent transformations (i.e. head-motion transform matrices, susceptibility distortion correction when available, and co-registrations to anatomical and output spaces). Gridded (volumetric) resamplings were performed using antsApplyTransforms (ANTs), configured with Lanczos interpolation to minimize the smoothing effects of other kernels (Lanczos 1964). Non-gridded

(surface) resamplings were performed using mri_vol2surf (FreeSurfer). First, a reference volume and its skull-stripped version were generated using a custom methodology of fMRIPrep. Susceptibility distortion correction (SDC) was omitted. The BOLD reference was then co-registered to the T1w reference using bbregister (FreeSurfer) which implements boundary-based registration (Greve and Fischl 2009). Co-registration was configured with six degrees of freedom. Head-motion parameters with respect to the BOLD reference (transformation matrices, and six corresponding rotation and translation parameters) are estimated before any spatiotemporal filtering using mcflirt (FSL 5.0.9, Jenkinson et al. 2002). BOLD runs were slice-time corrected to 0.45s (0.5 of slice acquisition range 0s-0.9s) using 3dTshift from AFNI 20160207 (Cox and Hyde 1997, RRID:SCR_005927). The BOLD time-series (including slice-timing correction when applied) were resampled onto their original, native space by applying the transforms to correct for head-motion. These resampled BOLD time-series will be referred to as preprocessed BOLD in original space, or just preprocessed BOLD. The BOLD time-series were resampled into standard space, generating a preprocessed BOLD run in MNI152NLin2009cAsym space. First, a reference volume and its skull-stripped version were generated using a custom methodology of fMRIPrep. Automatic removal of motion artifacts using independent component analysis (ICA-AROMA, Pruim et al. 2015) was performed on the preprocessed BOLD on MNI space time-series after removal of non-steady state volumes and spatial smoothing with an isotropic, Gaussian kernel of 6mm FWHM (full-width half-maximum). Corresponding "non-aggresively" denoised runs were produced after such smoothing. Additionally, the "aggressive" noise-regressors were collected and placed in the corresponding confounds file. Several confounding time-series were calculated based on the preprocessed BOLD: framewise displacement (FD), DVARS and three region-wise global signals. FD was computed using two formulations following Power (absolute sum of relative motions, Power et al. (2014)) and Jenkinson (relative root mean square displacement between affines, Jenkinson et al. (2002)). FD and DVARS are calculated for each functional run, both using their implementations in Nipype (following the definitions by Power et al. 2014). The three global signals are extracted within the CSF, the WM, and

the whole-brain masks. Additionally, a set of physiological regressors were extracted to allow for component-based noise correction (CompCor, Behzadi et al. 2007). Principal components are estimated after high-pass filtering the preprocessed BOLD time-series (using a discrete cosine filter with 128s cut-off) for the two CompCor variants: temporal (tCompCor) and anatomical (aCompCor). tCompCor components are then calculated from the top 2% variable voxels within the brain mask. For aCompCor, three probabilistic masks (CSF, WM and combined CSF+WM) are generated in anatomical space. The implementation differs from that of Behzadi et al. in that instead of eroding the masks by 2 pixels on BOLD space, the aCompCor masks are subtracted a mask of pixels that likely contain a volume fraction of GM. This mask is obtained by dilating a GM mask extracted from the FreeSurfer's aseg segmentation, and it ensures components are not extracted from voxels containing a minimal fraction of GM. Finally, these masks are resampled into BOLD space and binarized by thresholding at 0.99 (as in the original implementation). Components are also calculated separately within the WM and CSF masks. For each CompCor decomposition, the k components with the largest singular values are retained, such that the retained components' time series are sufficient to explain 50 percent of variance across the nuisance mask (CSF, WM, combined, or temporal). The remaining components are dropped from consideration. The head-motion estimates calculated in the correction step were also placed within the corresponding confounds file. The confound time series derived from head motion estimates and global signals were expanded with the inclusion of temporal derivatives and quadratic terms for each (Satterthwaite et al. 2013). Frames that exceeded a threshold of 0.5 mm FD or 1.5 standardised DVARS were annotated as motion outliers. All resamplings can be performed with a single interpolation step by composing all the pertinent transformations (i.e. head-motion transform matrices, susceptibility distortion correction when available, and co-registrations to anatomical and output spaces). Gridded (volumetric) resamplings were performed using antsApplyTransforms (ANTs), configured with Lanczos interpolation to minimize the smoothing effects of other kernels (Lanczos 1964). Non-gridded (surface) resamplings were performed using mri_vol2surf (FreeSurfer).

Many internal operations of fMRIPrep use Nilearn 0.6.2 (Abraham et al. 2014, RRID:SCR_001362), mostly within the functional processing workflow. For more details of the pipeline, see the section corresponding to workflows in fMRIPrep's documentation.

Copyright Waiver

The above boilerplate text was automatically generated by fMRIPrep with the express intention that users should copy and paste this text into their manuscripts unchanged. It is released under the CC0 license.

References

Abraham, Alexandre, Fabian Pedregosa, Michael Eickenberg, Philippe Gervais, Andreas Mueller, Jean Kossaifi, Alexandre Gramfort, Bertrand Thirion, and Gael Varoquaux. 2014. "Machine Learning for Neuroimaging with Scikit-Learn." Frontiers in Neuroinformatics 8. https://doi.org/10.3389/fninf.2014.00014.

Avants, B.B., C.L. Epstein, M. Grossman, and J.C. Gee. 2008. "Symmetric Diffeomorphic Image Registration with Cross-Correlation: Evaluating Automated Labeling of Elderly and Neurodegenerative Brain." Medical Image Analysis 12 (1): 26–41. https://doi.org/10.1016/j.media.2007.06.004.

Behzadi, Yashar, Khaled Restom, Joy Liau, and Thomas T. Liu. 2007. "A Component Based Noise Correction Method (CompCor) for BOLD and Perfusion Based fMRI." NeuroImage 37 (1): 90–101. https://doi.org/10.1016/j.neuroimage.2007.04.042.

Cox, Robert W., and James S. Hyde. 1997. “Software Tools for Analysis and Visualization of fMRI Data.” NMR in Biomedicine 10 (4-5): 171–78. https://doi.org/10.1002/(SICI)1099-1492(199706/08)10:4/5<171::AID-NBM453>3.0.CO;2-L.

Dale, Anders M., Bruce Fischl, and Martin I. Sereno. 1999. “Cortical Surface-Based Analysis: I. Segmentation and Surface Reconstruction.” NeuroImage 9 (2): 179–94. https://doi.org/10.1006/nimg.1998.0395.

Esteban, Oscar, Ross Blair, Christopher J. Markiewicz, Shoshana L. Berleant, Craig Moodie, Feilong Ma, Ayse Ilkay Isik, et al. 2018. “FMRIPrep.” Software. Zenodo. https://doi.org/10.5281/zenodo.852659.

Esteban, Oscar, Christopher Markiewicz, Ross W Blair, Craig Moodie, Ayse Ilkay Isik, Asier Erramuzpe Aliaga, James Kent, et al. 2018. “fMRIPrep: A Robust Preprocessing Pipeline for Functional MRI.” Nature Methods. https://doi.org/10.1038/s41592-018-0235-4.

Evans, AC, AL Janke, DL Collins, and S Baillet. 2012. “Brain Templates and Atlases.” NeuroImage 62 (2): 911–22. https://doi.org/10.1016/j.neuroimage.2012.01.024.

Fonov, VS, AC Evans, RC McKinstry, CR Almli, and DL Collins. 2009. “Unbiased Nonlinear Average Age-Appropriate Brain Templates from Birth to Adulthood.” NeuroImage 47, Supplement 1: S102. https://doi.org/10.1016/S1053-8119(09)70884-5.

Gorgolewski, K., C. D. Burns, C. Madison, D. Clark, Y. O. Halchenko, M. L. Waskom, and S. Ghosh. 2011. “Nipype: A Flexible, Lightweight and Extensible Neuroimaging Data Processing Framework in Python.” Frontiers in Neuroinformatics 5: 13. https://doi.org/10.3389/fninf.2011.00013.

Gorgolewski, Krzysztof J., Oscar Esteban, Christopher J. Markiewicz, Erik Ziegler, David Gage Ellis, Michael Philipp Notter, Dorota Jarecka, et al. 2018. “Nipype.” Software. Zenodo. https://doi.org/10.5281/zenodo.596855.

Greve, Douglas N, and Bruce Fischl. 2009. “Accurate and Robust Brain Image Alignment Using Boundary-Based Registration.” NeuroImage 48 (1): 63–72. https://doi.org/10.1016/j.neuroimage.2009.06.060.

Jenkinson, Mark, Peter Bannister, Michael Brady, and Stephen Smith. 2002. “Improved Optimization for the Robust and Accurate Linear Registration and Motion Correction of Brain Images.” NeuroImage 17 (2): 825–41. https://doi.org/10.1006/nimg.2002.1132.

Klein, Arno, Satrajit S. Ghosh, Forrest S. Bao, Joachim Giard, Yrjö Häme, Eliezer Stavsky, Noah Lee, et al. 2017. “Mindboggling Morphometry of Human Brains.” PLOS Computational Biology 13 (2): e1005350. https://doi.org/10.1371/journal.pcbi.1005350.

Lanczos, C. 1964. “Evaluation of Noisy Data.” Journal of the Society for Industrial and Applied Mathematics Series B Numerical Analysis 1 (1): 76–85. https://doi.org/10.1137/0701007.

Power, Jonathan D., Anish Mitra, Timothy O. Laumann, Abraham Z. Snyder, Bradley L. Schlaggar, and Steven E. Petersen. 2014. “Methods to Detect, Characterize, and Remove Motion Artifact in Resting State fMRI.” NeuroImage 84 (Supplement C): 320–41. https://doi.org/10.1016/j.neuroimage.2013.08.048.

Pruim, Raimon H. R., Maarten Mennes, Daan van Rooij, Alberto Llera, Jan K. Buitelaar, and Christian F. Beckmann. 2015. “ICA-AROMA: A Robust ICA-Based Strategy for Removing Motion Artifacts from fMRI Data.” NeuroImage 112 (Supplement C): 267–77. https://doi.org/10.1016/j.neuroimage.2015.02.064.

Satterthwaite, Theodore D., Mark A. Elliott, Raphael T. Gerraty, Kosha Ruparel, James Loughead, Monica E. Calkins, Simon B. Eickhoff, et al. 2013. “An improved framework for confound regression and filtering for control of motion artifact in the preprocessing of resting-state functional connectivity data.” NeuroImage 64 (1): 240–56. https://doi.org/10.1016/j.neuroimage.2012.08.052.

Tustison, N. J., B. B. Avants, P. A. Cook, Y. Zheng, A. Egan, P. A. Yushkevich, and J. C. Gee. 2010. “N4ITK: Improved N3 Bias Correction.” IEEE Transactions on Medical Imaging 29 (6): 1310–20. https://doi.org/10.1109/TMI.2010.2046908.

Zhang, Y., M. Brady, and S. Smith. 2001. “Segmentation of Brain MR Images Through a Hidden Markov Random Field Model and the Expectation-Maximization Algorithm.” IEEE Transactions on Medical Imaging 20 (1): 45–57. https://doi.org/10.1109/42.906424.
